# A multicountry perspective on gender differences in time use during COVID-19

**DOI:** 10.1073/pnas.2018494118

**Published:** 2021-03-08

**Authors:** Laura M. Giurge, Ashley V. Whillans, Ayse Yemiscigil

**Affiliations:** ^a^Department of Organizational Behavior, London Business School, London NW1 4SA, United Kingdom;; ^b^Negotiation, Organizations & Markets Unit, Harvard Business School, Boston, MA 02163;; ^c^Human Flourishing Program, Harvard University, Cambridge, MA 02138

**Keywords:** time, subjective well-being, gender, work–life balance, COVID-19

## Abstract

The COVID-19 pandemic has fundamentally altered how people spend time, with possible consequences for subjective well-being. Using diverse samples from the United States, Canada, Denmark, Brazil, and Spain (*n* = 31,141), following a preregistered analytic plan, and employing both mega- and meta-analyses, we find consistent gender differences in time spent on necessities. During the pandemic, women—especially mothers—spent more time on tasks such as childcare and household chores. To the extent that women spent more time on chores than men, they reported lower happiness. These data represent one of the most rigorous investigations of gender differences in time use during the forced lockdowns created by the COVID-19 pandemic, and point toward individual differences that should be considered when designing policies now and post–COVID-19.

## Significance

We find pervasive gender differences in time use during COVID-19. Surveys of diverse samples with over 30,000 respondents reveal that women—especially mothers—spent more time on necessities such as childcare and chores. In turn, time spent completing household chores was linked to lower well-being. This research reveals persistent time-use differences between women and men in household responsibilities during the COVID-19 pandemic.

The COVID-19 pandemic continues to disrupt our lives. Organizational leaders and policymakers are responding to the crisis by introducing new policies such as allowing employees to work from home until 2022 and switching to a hybrid organizational structure where employees can work some days at the office and some days at home (1, 2). However, these changes are being implemented with little robust empirical evidence regarding the nature and magnitude of the disruptions that people have been experiencing in their daily lives.

Most research so far has been devoted to how the pandemic has altered employee productivity (3, 4). A survey of 4,535 principal investigators revealed that female scientists with young children living at home experienced a decline in time spent on research (5). Analyzing patterns in technology use from over 3 million users, DeFilippis et al. (3) found that time spent in meetings decreased while the average workday expanded, with more time spent answering emails. As this research suggests, COVID-19 has transformed how people spend their time. Yet, no empirical research has examined time use beyond productivity. In this paper, we sought to understand how different groups of people across countries spent their time during the pandemic. We also examined whether any observed time-use differences predict differences in subjective well-being (SWB).

SWB refers to a person’s global evaluation of how happy they are and includes both a cognitive (i.e., assessments of one’s life quality) and an emotional component (i.e., high positive affect, low negative affect) (6). Recent research has started to investigate the relationship between time use and happiness and how this relationship depends on factors such as wealth and other demographic characteristics (7–10). Spending time on active leisure activities such as socializing or exercising can promote happiness (11). However, certain groups in society, primarily low-income women, tend to spend most of their time on necessities (e.g., household chores and caretaking responsibilities), leaving them “time-poor” and with little time for leisure activities (10).

COVID-19 provides a unique opportunity to study differences in time use and SWB for two primary reasons. First, recent estimates from the Gallup organization (12) suggest that the average number of days that people around the world have worked from home has more than doubled during the pandemic as compared with autumn 2019. This has likely resulted in many households having both household members working from home which should, in theory, equalize or at least reduce the gender gap in time spent on necessities between mothers and fathers. Second, recent estimates (13) suggest that in the United States alone the daily commute has saved working adults 89 million h each week since the pandemic started. Reduced commutes could also leave people with more time to engage in leisure activities. Thus, we might expect individuals to engage in more (vs. less) active leisure and to exhibit greater self-reported happiness as a result of this increase in leisure activities [see Smeets et al. (11) for a similar argument].

## Results

To explore the question of how people are spending their time, and whether and how time use is shaping SWB during COVID-19, we implemented nine surveys between mid-March and mid-June 2020, including nationally representative surveys of respondents living in the United States (*n* = 441) and Canada (*n* = 840), working parents living in the United States (*n* = 401), public sector workers living in Spain (*n* = 975), employed adults working from home in the United States (*n* = 1,518), Brazil (*n* = 21,874), and globally (*n* = 935), college students from Denmark (*n* = 3,233), and college students primarily studying in the United States (*n* = 924). We surveyed the US student sample again after 1 mo. See Table 1 for sample characteristics and *Materials and Methods* for details on the sampling strategy.

**Table 1.** Descriptive demographics of survey respondents and descriptive statistics for main variables across all samples

	Sample 1	Sample 2	Sample 3	Sample 4	Sample 5	Sample 6	Sample 7	Sample 8	Sample 9
Sample descriptions	US rep.	Canada rep.	US parents rep.	Spain working adults	US remote workers	Brazil remote workers	Remote workers global	Denmark college students	US college students
*N*	441	840	401	975	1,518	21,874	935	3,233	924
Mean (SD) age, y	49.27 (16.42)	42.58 (17.37)	38.01 (8.73)	47 (22.56)	42.69 (11.68)	43.18 (10.41)	39.10 (9.71)	26.22 (6.12)	21.00 (1.60)
White, %	79.4	56.0	–	–	61.1	59.2	65.9	–	62.0
Female, %	55.1	62.1	53.6	68.6	61.3	51.8	56.1	67.2	73.0
Parent, %	54.2	42.7	100.0	89.0	34.5	40.8	44.1		–
Master’s degree and above, %	–	–	–	43.6	57.9	46.8	62.2		<1
Employed full and part time, %	50.3	52.1	61.8	90.4	–	–	–		–
Mean (SD) weekly hours worked	–	–	–	37.16 (7.43)*	–	–	–		–
Median category, household income	$50K–$55K	6 (0; 10)	$70K–$80K	€3K–€5K^†^	$5K–$7K^†^	>R$7K^†^	Middle^†^^,^^‡^		$90K–$100K**
Median (range) no. children	1 (0; 6)	0 (0; 8)	2 (1; 7)	1 (0; 4)	0 (0; 7)	0 (0; 15)	0 (0; 9)		–
Married/partnership, %	57.2	60.1	82.8	85.9^§^	83.3^§^	85.6^§^	83.2^§^		95.6^§^
Mean (SD) life satisfaction	6.03 (2.53)	5.87 (2.62)	2.98 (1.16)^¶^	6.93 (1.47)	7.44 (1.81)	7.85 (1.99)	7.24 (1.95)	6.32 (2.13)	5.63 (1.92)
Mean (SD) positive affect	–	–	–	3.64 (9.74)	–	–	–	3.58 (0.74)	3.25 (0.75)
Mean (SD) negative affect	–	–	–	2.45 (0.89)	–	–	–	2.77 (0.87)	3.08 (0.84)

The symbol “–” indicates that the variable was not assessed in that sample. Sample size is based on all available data in each sample: Note that this differs slightly from our preregistration, where the sample size was based on all available data for our primary outcome variable: life satisfaction. Household income is reported in the local currency. rep., representative.

* Some respondents in this sample (32.2%) entered values that would be impossible on a weekly basis (e.g., 325 h). We imputed these values to reflect values that would be possible on a weekly basis (e.g., from 325 to 32.5 h).

^†^ In these studies, we recorded monthly income.

^‡^ In this sample, income was recorded from 1 = very low to 5 = very high.

^§^ In these studies, we measured whether respondents lived with at least one other adult (e.g., roommate, spouse, partner, parent, grandparent). The percentage reflects how many respondents indicated living with at least one other adult.

^¶^ Life satisfaction was measured as follows: “When compared to before the COVID-19 pandemic, how happy are you?” on a scale from 1 = much less happy to 5 = much happier.

** This refers to parents' household income.

We measured SWB across all samples by asking respondents to rate their overall life satisfaction. Respondents also reported how much time they allocated to various activities in a typical day during the pandemic. Our primary time-use outcomes included necessities, overall leisure, and work hours (11, 14). Time spent on necessities was typically a composite measure of household chores and taking care of others/family time. Overall leisure was a composite of active (e.g., exercising) and passive leisure (e.g., watching TV). Time spent working was a composite of time spent working for pay or studying (in student samples). See *SI Appendix*, Tables S1–S3 for detailed information on time-use measures in samples 1–8 and *SI Appendix*, Table S12*B* for sample 9.

As per our preregistered analytic plans (preregistration number 45781 for our cross-sectional samples: https://aspredicted.org/blind.php?x=e7qg3s; preregistration number 46013 for our longitudinal sample: https://aspredicted.org/blind.php?x=wf4d9u), we first examined how time use varied by sociodemographic groups (e.g., income, education, parental status, and relationship status or household size). Then, we looked at how SWB differed by sociodemographic group. Although we examined various sociodemographic groups as per our preregistration, the most reliable results we observed were differences in time use by gender, and differences in time use by gender and parental status. We therefore focus on these comparisons.

Based on comments that arose during peer review, we focus on describing the results of a mega-analysis conducted by pooling data from all nine samples. To allow for the inclusion of covariates, we also report the meta-analytic effects across samples with our preregistered covariates (*SI Appendix*). As per our preregistration, we report all sample-specific analyses in a separate file titled “Preregistered Sample-Specific Analyses” at the Open Science Framework (OSF): https://osf.io/cqr7k/?view_only=08c946a8ba2444e1ace32cccb28666d3.

### Mega- and Meta-Analytic Rationale.

Across all samples, we did not have a stopping rule for data collection given that we aimed to collect the largest number of respondents possible. Post hoc sensitivity analyses indicated that the effect sizes that we were powered to detect across samples with 80% confidence varied from *d* = 0.28 (sample 3, *n* = 401) to *d* = 0.03 (sample 6, *n* = 21,874) for differences in time use by gender, and from *d* = 0.26 (sample 1, *n* = 441) to *d* = 0.04 (sample 6, *n* = 21,874) for differences in time use by gender and parental status. See *SI Appendix*, Table S6 for detailed results, including sensitivity analyses with 85, 90, and 95% power. In light of the wide range of detectable effect sizes across samples, we pooled the data from all nine samples and conducted a mega-analysis. This analysis provided a sample size of 31,141 respondents with 56% female (*n* = 17,288) and 43% parents (*n* = 11,325). In these pooled data, we also had 58% mothers (*n* = 5,775) and 44% fathers (*n* = 5,419). Given that we had limited covariates that were measured consistently across samples and could be included in the mega-analysis, we further estimated meta-analytic effects that included our preregistered covariates for each sample (see *SI Appendix* for detailed results).

**Fig. 1.**
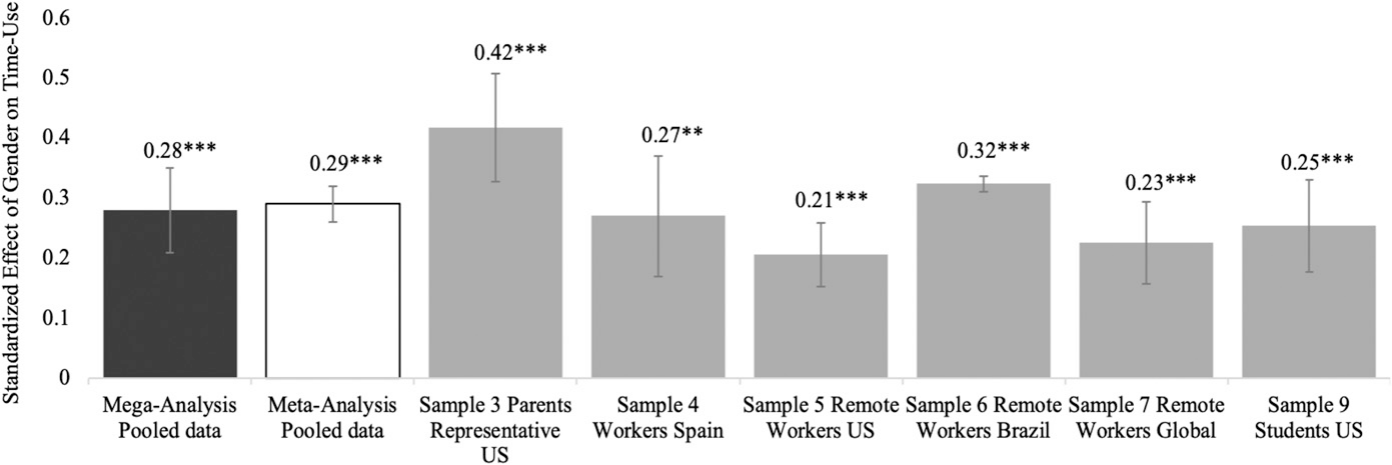
Differences in time spent on necessities by gender (1 = female) across samples. In samples 1, 2, and 8, we did not measure time spent on necessities. Covariates used in sample 1 were age, household income, employment status, marital status, and number of children. Covariates in sample 2 were age, employment status, marital status, number of children, and days since the survey was launched. Covariates in sample 3 were age, household income, employment status, marital status, and number of children. Covariates in sample 4 were age, household income, employment status, weekly work hours (apart from models with time-use work), household size, education level, number of children, and days since the survey was launched. Covariates in samples 5–7 were age, household income, household size, education level, number of children, and days since the survey was launched. Covariates in sample 8 were age and days since the survey was launched. Covariates in sample 9 were age, race, socioeconomic status (composite of parental education and income), household size, and days since the survey was launched. Error bars represent SEs. **P < 0.01, ***P < 0.001.

**Fig. 2.**
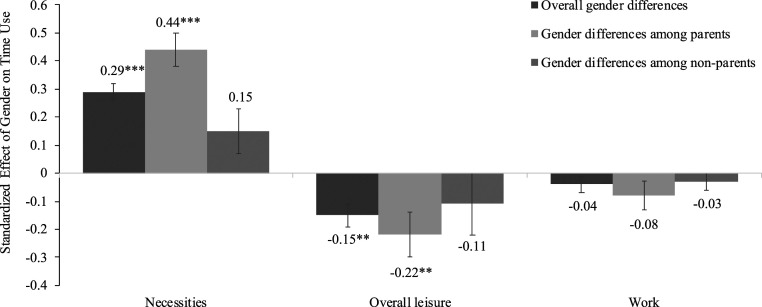
Time-use differences by gender (1 = female) and parental status from the meta-analysis. For the necessities (composite) effect, we included samples 4–7. For the overall leisure effect, we included samples 1 and 4–8. For the work effect, we included samples 1, 2, and 4–7. Covariates in sample 1 were age, household income, employment status, marital status, and number of children. Covariates in sample 2 were age, employment status, weekly work hours (apart for models with time-use work), marital status, number of children, and days since the survey was launched. Covariates in sample 3 were age, household income, employment status, marital status, and number of children. Covariates in sample 4 were age, household income, employment status, household size, education level, number of children, and days since the survey was launched. Covariates in samples 5–7 were age, household income, household size, education level, number of children, and days since the survey was launched. Covariates in sample 8 were age and days since the survey was launched. Covariates in sample 9 were age, race, socioeconomic status (composite of parental education and income), household size, and days since the survey was launched. Error bars represent SEs. **P < 0.01, ***P < 0.001.

Consistent with our preregistration, we examined gender differences in three time-use outcomes within each sample separately: 1) necessities, 2) overall leisure (active leisure + passive leisure), and 3) work (working for pay or studying). As shown in Fig. 1, we found consistent evidence that women spent more time on necessities compared to men during COVID-19. This result was supported by the mega-analysis (β = 0.28, 95% CI = [0.14, 0.42], *P* < 0.001). The meta-analytic results with covariates also showed notable gender differences for necessities (β = 0.29, 95% CI = [0.21, 0.36], *P* < 0.001). See Fig. 2 for the meta-analytic results of gender on overall leisure and work (see additional analyses and results in *SI Appendix*, Table S7*B*). There was heterogeneity for other time-use outcomes across samples (overall leisure, active leisure, passive leisure, and work) and no significant effect in the mega-analysis (*SI Appendix*, Table S11*B*). Thus, we do not discuss these results further in the main text (see *SI Appendix* for these analyses).

As per our preregistration, we tested whether gender differences in time use differed based on parental status. Sample-specific analyses of gender differences with parental status as a moderator can be found in *SI Appendix*, Table S8*A*. The mega-analysis found consistent moderation by parental status for gender differences in all time-use outcomes except for active leisure (*SI Appendix*, Table S11*C*). In these mega-analyses, parental status was a significant moderator of the relationship between gender and necessities (gender [1 = *female*] × parental status [1 = *parent*]: β = 0.25, 95% CI = [0.20, 0.29], *P* < 0.001), gender and overall leisure (gender [1 = *female*] × parental status [1 = *parent*]: β = −0.13, 95% CI = [−0.17, −0.08], *P* < 0.001), and gender and work (gender [1 = *female*] × parental status [1 = *parent*]: β = −0.10, 95% CI = [−0.15, −0.05], *P* < 0.001), such that gender differences in these activities were larger among parents vs. nonparents.

Among parents, there were significant gender differences for necessities (β = 0.48, 95% CI = [0.37, 0.60], *P* < 0.001) and work hours (β = −0.18, 95% CI = [−0.27, −0.08], *P* < 0.001). The effects for overall leisure approached conventional levels of significance (β = −0.15, 95% CI = [−0.29, 0.00], *P* = 0.052). Gender differences in time use were also evident when looking among nonparents for necessities (β = 0.23, 95% CI = [0.12, 0.35], *P* < 0.001) but not for work hours (β = −0.07, 95% CI = [−0.17, 0.02], *P* = 0.125) or overall leisure (β = −0.02, 95% CI = [−0.17, 0.13], *P* = 0.791). As per Fig. 2 and shown in detail in *SI Appendix*, Table S8*C*, the meta-analytic results showed that gender differences in time use were stronger for parents than nonparents, especially in time spent on necessities (gender differences among parents: β = 0.44, 95% CI = [0.25, 0.63], *P* < 0.001 vs. gender differences among nonparents: β = 0.15, 95% CI = [−0.10, 0.39], *P* = 0.058).

Complementing our analyses on time use, we also tested whether there were gender differences in happiness during COVID-19. Preregistered sample-specific analyses for happiness can be found in *SI Appendix*, Table S9*A*. Mega-analytic results showed no main effect of gender on happiness (β = −0.02, 95% CI = [−0.04, 0.00], *P* = 0.103). The mega-analysis also did not show a significant interaction by parental status in gender differences in happiness (gender [1 = *female*] × parental status [1 = *parent*]: β = −0.03, 95% CI = [−0.08, 0.02], *P* = 0.180). These results were similar in the meta-analysis (*SI Appendix*, Table S9*B*).

As per our preregistration, where relevant, our plan was to investigate the effects for the individual items that comprised our time-use composite measures, which led us to test the indirect effects for the two components of necessities, chores and caretaking/family time, in shaping the link between gender and happiness. In the mega-analysis, women spent more time in chores (β = 0.24, 95% CI = [0.14, 0.34], *P* < 0.001) and caretaking/family time (β = 0.22, 95% CI = [0.02, 0.41], *P* = 0.031), with consistent findings in the meta-analysis (*SI Appendix*, Table S7*B*). Consistent with our preregistration, we analyzed the relationship between time spent in chores and caretaking/family time and happiness (see *SI Appendix*, Table S10*A* for a sample-specific analysis). In the mega-analysis, while chores had a negative relationship with happiness (β = −0.06, 95% CI = [−0.10, −0.02], *P* = 0.006), caretaking/family time did not have a significant relationship (β = 0.04, 95% CI = [−0.04, 0.11], *P* = 0.315) (*SI Appendix*, Table S11*I*). The results were similar in the meta-analysis (*SI Appendix*, Table S10*B*).

**Fig. 3.**
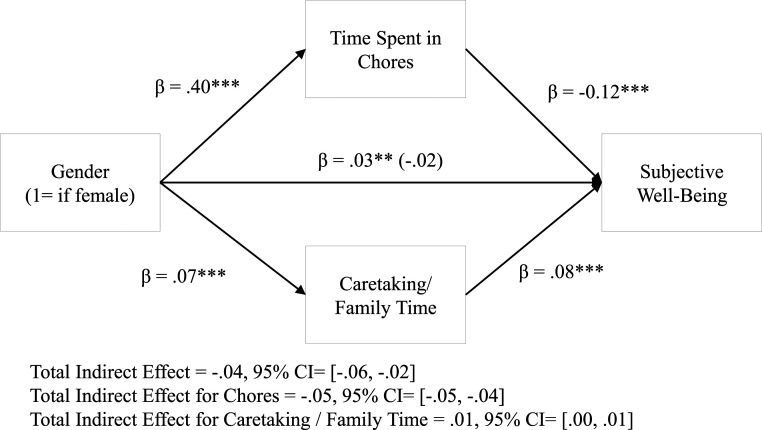
Effect of gender on SWB through time spent on chores and caretaking. All coefficients represent standardized betas and were estimated using generalized structural equation modeling with a random intercept. The beta in parentheses reports the effects of gender on subjective well-being without the mediators in the model. ***P < 0.001.

Motivated by these findings, we entered chores and caretaking simultaneously into the model described in Fig. 3. Using mega-analysis, we found a negative indirect effect on happiness through time spent completing chores (β = −0.05, 95% CI = [−0.05, −0.04], *P* < 0.001) and a small positive indirect effect through caretaking/family time in the full sample (β = 0.01, 95% CI = [0.00, 0.01], *P* < 0.001). Looking at parents only, we found similar results for chores (β = −0.07, 95% CI = [−0.08, −0.06], *P* < 0.001); yet, the positive effects via caretaking were no longer statistically significant (β = 0.00, 95% CI = [−0.00, 0.01], *P* = 0.218). Looking at nonparents, we found similar results for chores (β = −0.04, 95% CI = [−0.05, −0.03], *P* < 0.001); yet, the positive effects via caretaking were no longer significant (β = 0.00, 95% CI = [−0.00, 0.01], *P* = 0.812). These results were consistent controlling for age and employment status (*SI Appendix*, Table S11*E*). To the extent that women spent more time completing chores, they were more likely to report lower well-being.

### Sample 9: Longitudinal Analysis.

When looking at the samples as a whole, we did not observe gender differences in active leisure through the mega-analysis (β = −0.05, 95% CI = [−0.17, 0.07], *P* = 0.429) or meta-analysis (β = −0.08, 95% CI = [−0.24, 0.08], *P* = 0.229). However, our preregistered sample-specific analysis with covariates in sample 9 indicated a significant gender difference in active leisure between college-aged women and men (β = −0.03, 95% CI = [−0.04, −0.01], *P* = 0.001). In this sample, women reported lower levels of SWB as compared to men at time 1 (β = −0.23, 95% CI = [−0.36, −0.10], *P* < 0.001) and approximately 1 mo after the initial survey was implemented at time 2 (β = −0.16, 95% CI = [−0.29, −0.03], *P* = 0.013).*

In line with our preregistration, we examined whether differences in active leisure at time 1 accounted for these differences in SWB at time 2. Mediation analyses provided evidence that lower engagement in active leisure partly explained lower levels of SWB among women vs. men 1 mo later (β = −0.05, 95% CI = [−0.08, −0.02], *P* = 0.004). We did not find a significant indirect effect by necessities (β = 0.01, 95% CI = [−0.00; 0.03], *P* = 0.164), likely because this sample consisted of college students between the ages of 18 and 25 (*M*_age_ = 21). Indeed, our mega- and meta-analysis indicated that gender differences in necessities were more prominent for parents as compared with nonparents. See *SI Appendix* for all preregistered analyses with the longitudinal sample.

## Discussion

How did people spend their time during the outbreak of the COVID-19 pandemic and how did time use shape subjective well-being? Across eight cross-sectional surveys and one longitudinal survey, based on preregistered analyses (*n* = 31,141), we found consistent evidence that women spent more time on necessities compared to men. More specifically, women spent more time on household chores and caretaking tasks during COVID-19. These gender differences were stronger for parents. In a subsample of working adults (*n* = 24,327), we also measured respondents’ time use in a typical day prior to the pandemic. Exploratory analyses indicated a significant increase in time spent on necessities for women, and especially for mothers (*SI Appendix*, Fig. S6). Given that these are retrospective measures, more research is needed to explore whether there were fundamental shifts in the number of hours women spent on necessities.

In the full sample, to the extent that women (vs. men) spent more time completing chores, they reported lower levels of happiness. Similarly, to the extent that mothers (vs. fathers) spent more time on chores, they reported lower levels of happiness. These results point to the disproportionate burden that women, and especially mothers, experience in terms of time spent on necessities. It is possible that some of the happiness decreases among women that resulted from the additional provision of household chores is driven by the goal conflict of not being able to be an ideal employee and parent simultaneously (10, 15). Scholars should explore the psychological mechanisms underpinning why chore provision during COVID-19 undermines women’s happiness as well as potential interventions to alleviate the stress of household chores.

Our preregistered longitudinal analyses from sample 9 also found that young women reported lower well-being as compared to young men, in part due to differences in time spent in active leisure. Exploratory analyses with individual items that comprise the active leisure composite in sample 9 suggest that young women spent less time on hobbies (β = −0.32, *P* < 0.001; see *SI Appendix*, Table S12*I* for additional results). These data suggest that young females might be more negatively impacted by COVID-19 because they are engaging in less active leisure. More research is needed to understand the differential long-term effects of time use and happiness among students and other groups including older adults and healthcare workers who could be most vulnerable to lifestyle changes initiated by COVID-19.

Our analyses revealed no significant gender differences in subjective well-being in the pooled data (*n* = 31,141). Global surveys conducted prior to COVID-19 indicate that women typically experience marginally greater happiness as compared to men (16). These data suggest that the null result we observed may be indicative of a decrease in women’s happiness during COVID-19. In our college student data (sample 9), we found preliminary evidence in support of this claim. In this dataset, we asked respondents to indicate their happiness before and during COVID-19. In this study, women reported slightly but not significantly greater happiness than men before COVID-19 (β = 0.12, 95% CI = [−0.03, 0.27], *P* = 0.105) and significantly lower happiness than men during COVID-19 (β = −0.26, 95% CI = [−0.41, −0.12], *P* < 0.001). Longitudinal studies are needed to further investigate how COVID-19 has shaped gender differences in well-being.

It is also possible that we did not observe a significant main effect of gender on well-being because the negative consequences of additional chore provision were offset by completing these activities together or in the presence of other family members—which was more common during the COVID-19 lockdowns (17). Research based on extensive time-use data collected in the United Kingdom finds that people are happier when they are completing activities together with others (rather than alone) (18, 19). Initial exploratory analyses from sample 4 suggest that spending time together with one’s partner while completing household and caretaking tasks was associated with greater happiness (see *SI Appendix*, Table S15 for detailed results). Future research should explore how the immediate social context shapes time use and happiness during the pandemic.

## Materials and Methods

This project was approved by the Institutional Review Board at Harvard University (IRB20-0497 and IRB20-0476). All participants provided informed consent. We preregistered our analysis plan for the cross-sectional (https://aspredicted.org/blind.php?x=e7qg3s) and longitudinal data (https://aspredicted.org/blind.php?x=wf4d9u). Data and code are available through the OSF.

### Mega-Analyses.

We have pooled the data from all nine samples and conducted a mega-analysis to test differences in time use by gender and parental status (*SI Appendix*, Table S11 *B* and *C*). Since the data were clustered in nine different sampling groups, we used a random-intercept multilevel model where we allowed the intercept to vary for each group. Given that the covariates were not consistently measured across studies (*SI Appendix*, Table S1), we estimated the mega-analytic effects for our critical models without covariates. This allowed us to maintain a maximum sample size. Otherwise, the analysis would have dropped entire samples where certain covariates were not measured. The values of the dependent variables were standardized within each sample [see Enders and Tofighi (20) for recommendations to standardize the dependent variable in multilevel models] such that the within-sample mean values were 0 and SDs were 1.

Since our predictor of interest was a binary variable of gender (=1 if female), the beta coefficients can be interpreted as differences in time use between men and women in SD units. Following statistical recommendations (21), we compared the model fit between random-intercept and random-slope models using likelihood ratio tests and used the model with a better fit (*SI Appendix*, Table S11*A*). The rejection of the null (*P* value < 0.05) in the likelihood ratio tests we conducted led us to add the random slope to the random-intercept model to improve model fit for most models (for exceptions, see *SI Appendix*, Table S11*A*). Consistent with our preregistration, we conducted indirect effect and mediation analyses to examine whether time use predicted the effect of gender on SWB. In these analyses, we used random-intercept models due to convergence issues that are widely documented in complex random-slope models (21).

### Meta-Analyses.

Consistent with prior research (22), we estimated meta-analytic effects across the nine samples for the two primary analyses presented in the main text: 1) differences in time use by gender (*SI Appendix*, Table S7*B*), and 2) differences in time use by gender and parental status (*SI Appendix*, Table S8 *B* and *C*). We also estimated the meta-analytic effects for our secondary analyses: 1) differences in SWB by gender, and 2) differences in SWB by gender and parental status (*SI Appendix*, Table S9*B*). Given the large variation across samples, we estimated effect sizes via random-effects meta-analytic models (23) that assume the true effects vary among samples (24, 25). Similar to our approach in the mega-analysis, we standardized the outcome variables. Thus, the beta coefficients can be interpreted as differences in time use between men and women in SD units. As noted in the main text, the meta-analyses revealed similar effects to those observed in the mega-analyses. An overview of these effect sizes and the meta-analytic workbooks are available on our OSF page.

### Samples 1–9.

We surveyed 31,141^†^ individuals between mid-March and mid-June 2020 from different countries (e.g., Brazil, Spain, Denmark), including nationally representative samples (e.g., United States, Canada), and with diverse characteristics (e.g., working parents, remote workers, students). Below we provide details about each data collection. Here, we describe the regression analyses that we conducted in each sample, prior to pooling data for mega- and meta-analytic purposes.

Across all datasets, respondents rated their overall happiness on a scale from 0 (not at all) to 10 (extremely) (26). This served as our critical dependent measure. Single-item measures of well-being have been commonly used in prior large-scale survey research (27–29). In a subset of samples, we also measured positive and negative mood and combined these with the overall evaluation of happiness to create a SWB composite (28). See *SI Appendix*, Table S1 for more information about the measures employed in each sample. The primary time-use outcomes we focused on were as follows: work, overall leisure, active leisure, passive leisure, and necessities. Consistent with prior research (11, 14), we analyzed weighted statistics across samples, where the amount of time respondents reported spending on each activity was weighted by the total amount of time spent in all measured activities. Where available and in line with prior research (11), we split overall leisure into passive and active leisure. See *SI Appendix*, Tables S2 and S3 for sample time-use descriptive statistics for samples 1–8 and *SI Appendix*, Table S12*B* for sample 9.

Across all cross-sectional studies, we first examined how the primary time-use composites (i.e., work, overall leisure, necessities) varied by sociodemographic group, as available in each dataset: gender (1 = female), income (tertial split low vs. medium vs. high, unless otherwise specified), education (1 = at least a master’s degree), parental status (1 = yes), and relationship status (1 = married or in a marriage-like relationship). In studies where relationship status was not measured, we used household size (1 = living with others). For these analyses, we ran regression analyses and used Bonferroni corrections to control for the use of multiple comparisons. Next, we examined time-use differences in work, overall leisure, and necessities across groups by examining two-way interactions between sociodemographic groups. In these two-way interaction analyses, we treated income and education as continuous, consistent with past research (22).

Next, we examined how overall happiness differed by sociodemographic group (e.g., age, education) and by time use using regression analyses. As per our preregistration, if the sociodemographic variables were significantly associated with overall happiness, we ran indirect effect and mediation analyses to examine whether differences in the primary time-use composites explained why these sociodemographic variables predict overall happiness. Again, as per our preregistration, for sociodemographic variables that were not associated with overall happiness, we examined whether there was moderation such that people within a sociodemographic group who spent different amounts of time in the primary time-use composites reported different experiences of overall happiness. For these analyses, we again treated income and education as continuous.

Consistent with prior work (11, 22), we conducted the above analyses with and without these covariates as available per dataset (see Table 1 for sample demographics): age, gender, education, relationship status/household size, number of children, household income, employment status, and number of days since survey launch. In line with prior work (11), we examined passive leisure (e.g., activities such as watching TV, napping, resting) and active leisure (e.g., activities such as exercising, spending time with others) separately. All preregistered results with and without covariates based on sample-specific analyses are available in a separate file titled “Preregistered Sample-Specific Analyses” on the project’s OSF^‡^ page.

### Sample 1.

We recruited a representative sample of adults living in the United States. In this sample, respondents completed two items of life satisfaction. First, respondents answered the one item of overall happiness. Second, respondents completed the Cantril ladder (30) indicating where they currently stand in life on a ladder from 0 (bottom step = worst possible life) to 10 (top step = best possible life). Respondents further answered the one item of meaning in life (i.e., “To what extent do you agree that your life has a clear sense of purpose these days?”; 1 = strongly agree; 7 = strongly disagree). Next, respondents indicated how many hours per week they spent on paid work, active leisure, and passive leisure (see *SI Appendix*, Table S3*A* for detailed wording).

### Sample 2.

We recruited a representative sample of adults living in Canada. Respondents completed the same two items of life satisfaction and the one item of meaning in life as in sample 1. Next, respondents indicated how many hours per week they spent on paid work and active leisure (see *SI Appendix*, Table S3*B* for detailed wording). For samples 1 and 2, the data were collected as part of a larger, globally representative survey examining how cultural norms shaped COVID-19 outbreaks.

### Sample 3.

We recruited a representative sample of parents living in the United States as part of a larger nationally representative survey of childcare during COVID-19. In this sample, respondents completed one item of overall happiness anchored pre–COVID-19 (i.e., “When compared to before the COVID-19 pandemic, how happy are you?”; 1 = much less happy to 5 = much happier). Next, respondents indicated the percentage of total time in a typical day during the COVID-19 pandemic they allocated to various activities (see *SI Appendix*, Table S3*C* for detailed measures).

### Sample 4.

We recruited working adults living in Spain as part of a larger survey examining time use, work experiences, and well-being among public sector workers. Survey items were translated and backtranslated in Catalan. Respondents completed the one item of overall happiness and measures assessing the affective component of SWB. Respondents rated their positive and negative affect over the past 4 wk using the Schedule for Positive and Negative Affect scale [SPANE (28); 1 = very rarely/never to 5 = very often/always]. Respondents also rated their meaning in life over the past 4 wk using a three-item scale (31) (e.g., “I understand my life’s meaning”; 1 = not at all true to 7 = extremely true). Next, respondents indicated the percentage of their time in a typical workday since COVID-19 that they allocated to various activities (see *SI Appendix*, Table S3*D* for detailed measures).

### Sample 5.

We recruited working adults living in the United States as part of a larger data collection effort examining remote working during COVID-19. In this sample, respondents completed two items of life satisfaction. First, respondents answered the one item of overall happiness. Second, respondents answered a second item capturing overall satisfaction with life (i.e., “Overall, how satisfied are you with your life?”; 1 = not at all to 10 = completely). Respondents indicated how many hours they spent on various activities in a typical workday since working remotely due to COVID-19 (see *SI Appendix*, Table S3*E* for detailed measures) and how many hours they recalled spending on the same activities in a typical workday before COVID-19 (see *SI Appendix*, Table S13*A* for detailed results).

### Sample 6.

We recruited working adults living in Brazil as part of a larger survey examining remote working during COVID-19. Respondents completed the same items of life satisfaction as in sample 5. Next, respondents indicated how many hours they spent on various activities in a typical workday since working remotely due to COVID-19 (see *SI Appendix*, Table S3*F* for detailed measures) and how many hours they recalled spending on the same activities in a typical workday before COVID-19 (see *SI Appendix*, Table S13*B* for detailed results).

### Sample 7.

We recruited working adults globally as part of a larger survey examining remote working during COVID-19.^§^ Respondents completed the same items of life satisfaction as in samples 5 and 6. Next, respondents indicated how many hours they spent on various activities in a typical workday since working remotely due to COVID-19 (see *SI Appendix*, Table S3*G* for detailed measures) and how many hours they recall spending on the same activities in a typical workday before COVID-19 (see *SI Appendix*, Table S13*C* for detailed results).

### Sample 8.

We recruited postsecondary students living in Denmark as part of a larger survey examining time use, meaning, and well-being among students during COVID-19. Respondents completed the one item of overall happiness as well as the positive affect, negative affect, and meaning in life scales used in sample 4. Next, respondents indicated how many hours they spent during the previous week on various activities (see *SI Appendix*, Table S3*H* for detailed measures).

### Sample 9.

We supplemented these cross-sectional samples with a longitudinal survey examining time-use differences by sociodemographic group and how time use relates to SWB. This sample was collected as part of a larger survey examining students’ meaning, time use, and well-being at the outbreak of COVID-19. We advertised the time 1 (T1) survey during March to May 2020 to full-time students from US colleges between the ages of 18 and 25. Respondents who did not fit these criteria could not access the survey. We used online convenience sampling where the research team distributed the survey link in their networks and on social media. We asked initial respondents to refer the survey to their network. As compensation, we provided participants a 1-in-20 chance of winning a $50 Amazon gift card. Finally, we advertised our survey on Amazon Mechanical Turk (AMT), using the same screening criteria. Respondents on AMT were paid $3 for completing our survey. We contacted respondents who completed our T1 survey for our time 2 (T2) survey during May to June 2020 and used the same compensation for both data collection methods.

We collected 1,887 responses at T1. We identified unique responses using respondents’ self-generated aliases. When aliases were identical, we excluded the observation with a higher number of missing values. If the number of missing values was equal, we excluded the observation with a later response date—assuming that the earlier response could be considered to be “naive.” This resulted in 1,869 observations. At T2, we collected 1,210 responses. Applying the same exclusion criteria as above left us with 1,104 observations at T2 (59% of T1 responses after exclusions). In total, we were able to match 924 observations using aliases (49% of T1 responses after exclusions). The majority of our sample (81%) was recruited via online channels (vs. AMT).

Respondents completed the one item of overall happiness as well as the positive affect, negative affect, and meaning in life scales used in samples 4 and 8. In this sample, respondents provided more extensive descriptions of how they spent their time over the previous 7 d using items adapted from prior work on time use (11, 14). Work was measured as the sum of time spent in commuting, working, and school/learning. Active leisure was measured as the sum of time spent praying/worshipping/meditating, socializing, exercising, in intimate relations, going outdoors, and on hobbies. Passive leisure was measured as the sum of time spent watching TV, napping/resting, relaxing, and doing nothing. Necessities was measured as the sum of time spent shopping, on personal hygiene, preparing food, and doing housework. All measures were captured at T1 and T2.

## Supplementary Material

Supplementary File

## Data Availability

Data and code reported in this article have been deposited in the Open Science Framework (https://osf.io/cqr7k/?view_only=08c946a8ba2444e1ace32cccb28666d3).

## Footnotes

*Our preregistered analyses found positive links between active leisure and SWB in the mega-analysis (β = 0.12, 95% CI = [0.07, 0.18], *P* < 0.001), with consistent findings in the meta-analysis (*SI Appendix*, Table S10*B*).

^†^Our preregistration indicates 30,028 as the sample size because it reports the nonmissing, available data for our critical dependent variable: the overall happiness measure. Given that missing-data patterns differ for time use and happiness and the preregistered sample may change when examining time-use outcomes (without happiness), we now report all available data in the analysis which may involve missing data for happiness and time use. Note that we also included the baseline wave from the longitudinal student sample in the cross-sectional data for the mega-analysis in order to make use of all available data. The inclusion of this sample did not change the results in any meaningful way. See *SI Appendix*, Table S4 for sample sizes for nonmissing values across samples.

^‡^We preregistered running analyses with ideal time-use items where available (i.e., samples 4–7). However, given the primary focus in this paper is on understanding time use before and during COVID-19 across sociodemographic groups and how these time-use differences relate to SWB, we have no longer analyzed ideal time use.

^§^We preregistered nine datasets; however, we combined two datasets that used the same measures, one where we recruited remote workers living in Israel (*n* = 231) with one where we recruited remote workers globally (*n* = 702), to have a higher sample size and thus greater statistical power.
