# Supplementary material for: A multicountry perspective on gender differences in time use during COVID-19
Source: Proc Natl Acad Sci U S A. 2021 Mar 8;118(12):e2018494118. doi: 10.1073/pnas.2018494118 (PMC7999869; doi:10.1073/pnas.2018494118)
Supplement: Supplementary File [file pnas.2018494118.sapp.pdf]

Supporting Information for

A Multi-Country Perspective on Gender Differences in Time-Use During COVID-19

This document contains:

Detailed Measures and Demographics Across Sample 1-8 2

Detailed Time-Use Measures and Descriptives Across Samples 1-8 3

Sample size for Nonmissing Values Across Samples 7

Measurement of Necessities Time-Use Composite Across Samples 8

Sensitivity Analyses for Effects of Gender and Gender X Parental Status Across Samples 9

Meta-Analyses: Gender Differences in Time-Use Across Samples (with Covariates) 11

Individual and Meta-Analytic Effect Sizes of Gender Differences in Time-Use Across Samples (with Covariates) 14

Meta-Analyses: Gender X Parental Status Differences in Time-Use Across Samples (with Covariates) 23

Meta-Analyses: Gender and Gender X Parental Status Differences in Happiness Across Samples (with Covariates) 28

Meta-Analyses: Time-Use and Happiness Across Samples (with Covariates) 31

Mega-Analyses Results 33

Sample 9 (924): All Results for Longitudinal Sample 42

Exploratory analyses with recalled pre-COVID-19 time-use 51

Additional Analyses Sample 4: Shared Time on Chores and Caretaking Responsibilities 61

Data, code, meta-analytic workbooks, and analyses with the individual samples for this paper are available at: https://osf.io/cqr7k/?view_only=08c946a8ba2444e1ace32cccb28666d3.

Our preregistered analytic plan for the correlational data is available at: http://aspredicted.org/blind.php?x=e7qg3s

Our preregistered analytic plan for the longitudinal data is available at: http://aspredicted.org/blind.php?x=wf4d9u

Detailed Measures and Demographics Across Sample 1-8

Table S1

Additional information for measures and demographic variables in Studies 1-8

Study	*N*	Description	Primary Well-Being Measures	Primary Time-Use Measures	Secondary Time-Use Measures	Secondary Well-Being Measures	Omitted Demographics [a]
1	441	US Representative [c]	2-item SWL<br>(α = .78)	1-item Work<br>1-item Active leisure<br>1-item Passive leisure	--	1-item Purpose in Life	Education<br># of weekly work hours
2	840	Canada Representative [d]	2-item SWL<br>(α = .78)	1-item Work<br>1-item Overall leisure	--	1-item Purpose in Life	Education<br>Income<br># of weekly work hours
3	401	US parents Representative [e]	1-item SWL	1-item Work<br>1-item Necessities<br>2-item Active leisure	Sleep<br>Other	--	Education<br>Parental status<br># of weekly work hours
4	975	Spain working adults	3-item SWL<br>(α = .80)	2-item Work<br>3-item Active leisure<br>1-item Passive leisure<br>2-item Necessities	Sleep<br>Other	3-item Meaning in life<br>(α = .87)	Marital status [c]
5	1,518	US remote workers	2-item SWL<br>(α = .89)	2-item Work<br>2-item Active leisure<br>1-item Passive leisure<br>1-item Necessities	Sleep	--	Employment status<br>Marital status [c]<br># of weekly work hours
6	21,874	Brazil remote workers	2-item SWL<br>(α = .89)	2-item Work<br>2-item Active leisure<br>1-item Passive leisure<br>1-item Necessities	Sleep	--	Employment status<br>Marital status [c]<br># of weekly work hours
7	935	Other countries remote workers	2-item SWL<br>(α = .85)	2-item Work<br>2-item Active leisure<br>1-item Passive leisure<br>1-item Necessities	Sleep	--	Employment status<br>Marital status [c]<br># of weekly work hours
8	3,233	Danish post-secondary students	3-item SWL<br>(α = .85)	2-item work<br>1-item Active leisure<br>1-item Passive leisure	--	3-item Meaning in life<br>(α = .90)	Income<br>Education<br>Marital status<br>Parental status<br>Employment status<br># of weekly work hours

Note. The symbol "--" indicates that the variable was not assessed in that study. [a] Across all studies (unless otherwise indicated as omitted), respondents reported their age and gender. [b] In these studies we recorded household size (i.e. whether respondents live alone or with others in the household). [c] Respondents were nationally representative in terms of age, gender, ethnicity, and occupation status (i.e., full-time employed) for the US. [d] Respondents were nationally representative in terms of age, gender, ethnicity, and occupation status (i.e., full-time employed) for Canada. [e] Respondents were nationally representative in terms of age, gender and ethnicity. Sample size is based on all available data in each sample.

Detailed Time-Use Measures and Descriptives Across Samples 1-8

Table S2
Descriptive statistics for the primary time-use measures in studies 1-8

	Sample 1	Sample 2	Sample 3	Sample 4	Sample 5	Sample 6	Sample 7	Sample 8
**Time-Use Composites**	US representative [a d]	Canada representative [a d]	US parents representative	Spain working adults [c]	US remote workers [d]	Brazil remote workers [d]	Other countries remote workers [d]	Denmark college students [d]
Work	27.37% (30.16)	40.22% (40.04)	16.90% (15.53)	34.84% (15.07)	38.29% (12.43)	34.94% (9.42)	35.95% (9.73)	46.33% (22.24)
Overall leisure [b]	72.63% (30.16)	59.96% (40.01)	25.00% (15.57)	21.67% (12.61)	15.99 (6.99)	14.62% (6.81)	15.80% (7.43)	53.70% (22.23)
Active leisure	22.72% (19.71)	--	13.57% (11.87)	12.45% (9.53)	6.75 (4.22)	5.00% (4.21)	5.86% (4.54)	22.24% (16.29)
Passive leisure	50.14% (27.82)	--	--	9.22% (7.62)	9.38 (5.68)	9.78% (5.39)	10.06% (5.87)	31.48% (19.65)
Necessities	--	--	35.67% (19.78)	21.07% (14.32)	16.47 (9.34)	20.87 (10.27)	18.31% (10.14)	--

Note. The symbol “--" indicates that the variable was not assessed in that study. Descriptive statistics for the time-use measures are reported as episode-weighted statistics (i.e. the percentage of time that respondents reported spending on each activity is weighted by the total amount of time they spent in all other activities measured within that sample). [a] In these studies we measured time-use on work, active, and passive leisure with 1-item. [b] Overall leisure is a composite of active and passive leisure across all studies. The composites differ per study and the exact items are presents in the tables below [c] Passive leisure in these studies was measured with 1-item only. [d] In these datasets, active and passive leisure were measured with 1-item each.

Table S3a

Summary of individual time-use activities (Sample 1)

**Time-use Composites**	**Individual Time-Use Items (# of hours per week)**	**Mean (*SD*)**
Work	# of hours per week spent on paid work	16.56 (19.41)
Active leisure	# of hours per week spent on active leisure (e.g., going outdoors, praying/meditating, exercising, intimate relations, socializing)	24.91 (22.36)
Passive leisure	# of hours per week spent on passive leisure (e.g., watching TV, relaxing, doing nothing, or resting)	38.65 (55.54)

Note. *N* = 440. *SD* = Standard deviation. In this dataset, we did not record times spent on necessities.

Table S3b

Summary of individual time-use activities (Sample 2)

**Time-use Composites**	**Individual Time-Use Items (# of hours per week)**	**Mean (*SD*)**
Work	# of hours per week spent on paid work	16.21 (19.68)
Active leisure	# of hours per week spent on active leisure (e.g., going outdoors, praying/meditating, exercising, intimate relations, socializing)	15.08 (56.87)

Note. *N* = 840. *SD* = Standard deviation. In this dataset, we did not record time spent on passive leisure or necessities.

Table S3c

Summary of individual time-use activities (Sample 3)

**Time-use Composites**	**Individual Time-Use Items (% out of 100)**	**Mean (*SD*)**
Work	% Working on tasks for one’s employer/company	16.90 (15.53)
Overall leisure	% Taking care of yourself (exercising, meditating, watching TV, reading, relaxing, etc.)	11.43 (9.94)
Active leisure	% Spending time alone with your partner	7.41 (7.57)
	% Socializing with others (e.g., talking in-person or virtually with your friends and family)	6.16 (7.46)
Necessities	% Doing household chores (e.g., preparing meals, doing laundry, cleaning, etc.)	12.13 (9.81)
	% Taking care of your children (e.g., homeschooling, reading, playing with them, etc.)	23.54 (16.70)
Other activities	% Sleeping	20.86 (13.71)
	% Other	1.57 (6.65)

Note. *SD* = Standard deviation. In this dataset, we did not record passive leisure separately from overall leisure.

Table S3d

Summary of individual time-use activities (Sample 4)

Time-use Composites	Individual Time-Use Items (% out of 100)	Mean (*SD*)
Work	% Paid work (e.g., doing tasks for your employer/company)	28.43 (14.76)
	% Deep work hours (hours in which you work continuously on one core work task for extended periods of time)	6.41 (11.96)
Active leisure	% Leisure activities like going outdoors, praying/socializing, intimate relations, socializing, volunteering	6.68 (6.24)
	% Social activities with work colleagues (e.g., virtual drinks)	1.26 (2.72)
	% Social activities with friends and family (e.g., virtual get together)	4.52 (5.00)
Passive leisure	% Leisure activities like watching TV, relaxing, doing nothing, resting, etc.	9.22 (7.62)
Necessities	% Doing household chores (e.g., preparing meals, doing laumdry, cleaning, etc.)	10.93 (6.24)
	% Taking care of others in the household (e.g., your kids, elderly, etc.)	10.14 (12.36)
Other activities	% Sleeping	21.65 (10.70)
	% Other	.78 (3.18)

Note. *SD* = Standard deviation.

Table S3e

Summary of individual time-use activities (Sample 5)

Time-use Composites	Individual Time-Use Items (# hours)	Mean (*SD*)
Work	Working productively (uninterrupted work time)	5.80 (2.18)
	Working unproductively (distracted work time/multi-tasking)	2.68 (1.97)
Active leisure	Leisure activities like exercising, playing games, socializing, going outdoors	1.60 (1.26)
Passive leisure	Leisure activities like watching TV, relaxing, doing nothing, resting	2.26 (1.59)
Necessities	Doing errands or housework	1.63 (1.30)
	Taking care of or spending time with family [a]	2.53 (2.36)
Other activities	Sleeping	6.89 (1.96)

Note. *SD* = Standard deviation. [a] This item can be treated as both active leisure (spending time with family) and a necessity (taking care of family). We treat it as a necessity given that COVID-19 has likely placed additional demands on people's time, including the need to spend more time with family that is not necessarily viewed as leisure time.

Table S3f

Summary of individual time-use activities (Sample 6)

**Time-use Composites**	**Individual Time-Use Items (# hours)**	**Mean (*SD*)**
Work	Working productively (uninterrupted work time)	5.39 (2.17)
	Working unproductively (distracted work time/multi-tasking)	3.24 (2.46)
Active leisure	Leisure activities like exercising, playing games, socializing, going outdoors	1.28 (1.25)
Passive leisure	Leisure activities like watching TV, relaxing, doing nothing, resting	2.48 (1.62)
Necessities	Doing errands or housework	2.40 (1.79)
	Taking care of or spending time with family [a]	3.14 (2.66)
Other activities	Sleeping	7.14 (1.26)

Note. *SD* = Standard deviation. [a] This item can be treated as both active leisure (spending time with family) and a necessity (taking care of family). We treat it as a necessity given that COVID-19 has likely placed additional demands on people's time, including the need to spend more time with family that is not necessarily viewed as leisure time.

Table S3g

Summary of individual time-use activities (Sample 7)

**Time-use Composites**	**Individual Time-Use Items (# hours)**	**Mean (*SD*)**
Work	Working productively (uninterrupted work time)	5.54 (2.50)
	Working unproductively (e.g., distracted work time/multi-tasking)	3.20 (2.24)
Active leisure	Leisure activities like exercising, playing games, socializing, going outdoors	1.51 (1.48)
Passive leisure	Leisure activities like watching TV, relaxing, doing nothing, resting	2.58 (1.87)
Necessities	Doing errands or housework	2.09 (1.95)
	Taking care of or spending time with family [a]	2.86 (2.58)
Other activities	Sleeping	7.20 (1.35)

Note. *SD* = Standard deviation. [a] This item can be treated as both active leisure (spending time with family) and a necessity (taking care of family). We treat it as a necessity given that COVID-19 has likely placed additional demands on people's time, including the need to spend more time with family that is not necessarily viewed as leisure time.

Table S3h

Summary of individual time-use activities (Sample 8)

**Time-use Composites**	**Individual Time-Use Items (# hours)**	**Mean (*SD*)**
Work	Schoolwork	29.14 (18.88)
	Professional work	7.52 (11.72)
Active leisure	Leisure activities like going outdoors, etc.	19.01 (17.36)
Passive leisure	Leisure activities like watching TV, etc.	26.86 (21.32)

Note. *SD* = Standard deviation. In this study we did not record necessities.

Sample size for Nonmissing Values Across Samples

Table S4

Sample size for nonmissing values

	**Sample 1**	**Sample 2**	**Sample 3**	**Sample 4**	**Sample 5**	**Sample 6**	**Sample 7**	**Sample 8**	**Sample 9**	**Total**
Happiness	440	840	400	866	1,513	21,844	933	3,182	924	30,942
Necessities	-	-	401	710	1,499	21,792	926	-	921	26,249
Overall leisure	438	783	401	710	1,501	21,773	928	2,799	921	30,254
Work	438	782	401	710	1,507	21,834	928	2,799	921	30,320
Active Leisure	438	783	401	710	1,492	21,140	916	2,798	921	29,599
Passive Leisure	436	-	-	710	1,486	21,717	924	2,798	921	28,992
Chores	-	-	401	710	1,494	21,652	923	-	914	26,094
Caretaking/Family Time	-	-	401	710	1,448	21,406	912	-	-	24,877
Gender	441	833	401	880	1,459	21,560	910	3,229	899	30,612
Parental Status	441	840	22,798	525	1,518	21,871	933	-	-	48,926
Employment Status	441	840	401	884	1,518	21,874	935	3,233	3,233	33,359
Age	441	840	399	883	1,446	21,745	909	3,233	924	30,820

*Note.*Happiness was measured as a single-item question: "Taking all things together, how happy would you say you are? 0 = not at all to 10 = extremely" in Samples 4 to 9; "In general, to what extent do you feel happy these days? 0 = very unhappy to 10 = very happy" in Samples 1 to 3; and "When compared to before the COVID-19 pandemic, how happy are you? 1 = much less happy to 5 = much more happy". Tables S3a to S3e describes the sample specific items that were used to calculate the time-use composites.

Measurement of Necessities Time-Use Composite Across Samples

Table S5
Measurement of Necessities Across Samples

Sample	Measurement of Necessities	Measurement of Chores	Measurement of Taking Care of Others
**Sample 1 Representative US**	NA	NA	NA
**Sample 2 Representative Canada**	NA	NA	NA
**Sample 3 Parents - Representative US**	Taking care of your [Field-who] (e.g., homeschooling, reading, playing with them, etc.)<br>Doing household chores (e.g., preparing meals, doing laundry, cleaning, etc.)	Doing household chores (e.g., preparing meals, doing laundry, cleaning, etc.)	Taking care of your [Field-who] (e.g., homeschooling, reading, playing with them, etc.)
**Sample 4 Workers Spain**	Doing household chores (e.g., preparing meals, doing laundry, cleaning, etc.):<br>Taking care of others in the household (e.g. your kids, elderly, etc.	Doing household chores (e.g., preparing meals, doing laundry, cleaning, etc.)	Taking care of others in the household (e.g. your kids, elderly, etc.
**Sample 5 Remote Workers US**	Doing errands or housework<br>Taking care of or spending time with family	Doing errands or housework	Taking care of or spending time with family
**Sample 6 Remote Workers Brazil**	Doing errands or housework<br>Taking care of or spending time with family	Doing errands or housework	Taking care of or spending time with family
**Sample 7 Remote Workers Global**	Doing errands or housework<br>Taking care of or spending time with family	Doing errands or housework	Taking care of or spending time with family
**Sample 8 Students Denmark**	NA	NA	NA
**Sample 9 Students US**	Time spent shopping, personal hygiene, preparing food, doing housework.	Preparing food, doing housework	NA

Sensitivity Analyses for Effects of Gender and Gender X Parental Status Across Samples

Table S6a

Sensitivity Analyses for Gender Effects Across Samples

	**Sample size**	**95% Power**	**90% Power**	**85% Power**	**80% Power**
Sample 1	441 (men = 196 vs. women = 243)	d = .34	d = .31	d = .28	d = .26
Sample 2	840 (men = 311 vs. women = 522)	d = .25	d = .23	d = .21	d = .20
Sample 3	401 (men = 185 vs. women = 215)	d = .36	d = .32	d = .30	d = .28
Sample 4	975 (men = 274 vs. women =606)	d = .26	d = .23	d = .21	d = .20
Sample 5	1,518 (men = 560 vs. women = 895)	d = .19	d = .17	d = .16	d = .15
Sample 6	21,874 (men = 10387 vs. women = 11173)	d = .04	d = .04	d = .04	d = .03
Sample 7	935 (men = 398 vs. women = 512)	d = .24	d = .21	d = .20	d = .18
Sample 8	3,233 (men = 769 vs. women = 2460)	d = .16	d = .13	d = .12	d = .11
Sample 9	924 (men = 241 vs. women = 658)	d = .27	d = .24	d = .23	d = .21

Note. These sensitivity analyses illustrate the effect size (Cohen’s d) we would be able to detect given each sample size at α = .05.

Table S6b
Sensitivity Analyses for Gender X Parental Status Interaction Effects Across Samples

	Sample size	95% Power	90% Power	85% Power	80% Power
Sample 1	N_{total} = 441	d = .34	d = .30	d = .28	d = .26
	$N_{\text{Non-Dads}}$ = 95 vs. $N_{\text{Non-Moms}}$ = 107	d = .51	d = .46	d = .42	d = .40
	N_{Dads} = 103 vs. N_{Moms} = 136	d = .47	d = .43	d = .39	d = .37
Sample 2	N_{total} = 840	d = .24	d = .27	d = .25	d = .22
	$N_{\text{Non-Dads}}$ = 167 vs. $N_{\text{Non-Moms}}$ = 311	d = .35	d = .31	d = .29	d = .27
	N_{Dads} = 144 vs. N_{Moms} = 211	d = .39	d = .35	d = .32	d = .30
Sample 4	N_{total} = 975	d = .27	d = .25	d = .23	d = .22
	$N_{\text{Non-Dads}}$ = 22 vs. $N_{\text{Non-Moms}}$ = 36	d = .99	d = .89	d = .83	d = .77
	N_{Dads} = 147 vs. N_{Moms} = 319	d = .36	d = .32	d = .30	d = .28
Sample 5	N_{total} = 1,518	d = .22	d = .20	d = .18	d = .17
	$N_{\text{Non-Dads}}$ = 340 vs. $N_{\text{Non-Moms}}$ = 613	d = .24	d = .22	d = .20	d = .19
	N_{Dads} = 220 vs. N_{Moms} = 286	d = .32	d = .29	d = .27	d = .25
Sample 6	N_{total} = 21,874	d = .05	d = .05	d = .04	d = .04
	$N_{\text{Non-Dads}}$ = 5947 vs. $N_{\text{Non-Moms}}$ = 6788	d = .06	d = .06	d = .05	d = .05
	N_{Dads} = 4438 vs. N_{Moms} = 4384	d = .08	d = .07	d = .06	d = .06
Sample 7	N_{total} = 935	d = .28	d = .25	d = .24	d = .22
	$N_{\text{Non-Dads}}$ = 216 vs. $N_{\text{Non-Moms}}$ = 287	d = .33	d = .29	d = .27	d = .25
	N_{Dads} = 181 vs. N_{Moms} = 224	d = .36	d = .32	d = .30	d = .28

Note. These sensitivity analyses illustrate the effect size (Cohen's d) we would be able to detect given each sample size at α = .05. In sample 3 we collected data among working parents. In Samples 8-9, we collected data among students and did not measure parental status.

Meta-Analyses: Gender Differences in Time-Use Across Samples (with Covariates)

Table S7a

Standardized beta and r-partial for gender (1=female) predicting time-use (with covariates)

	**Necessities**			**Chores**			**Caretaking/ Family time**			**Overall leisure**			**Active leisure**			**Passive leisure**			**Work**		
**Sample**	β (*p*-value)	(se)	r	β (*p*-value)	(se)	r	β (*p*-value)	(se)	r	β (*p*-value)	(se)	r	β (*p*-value)	(se)	r	β (*p*-value)	(se)	r	β (*p*-value)	(se)	r
**1**										.05 (.417)	.05	.04	.03 (.719)	.09	.02	.04 (.584)	.08	.03	-.05 (.417)	.06	-.04
**2**													.14 (.006)	.05	.10				-.13 (.010)	.05	-.09
**3**	.42 (<.001)	.09	.23	.12 (.271)	.11	.06	.43 (<.001)	.09	.22	-.26 (.016)	.11	-.26	-.32 (.003)	.11	-.15				-.08 (.326)	.09	-.05
**4**	.27 (.009)	.10	.13	08 (.460)	.11	.04	.27 (.008)	.10	.13	-23 (.014)	.09	-.12	-.10 (.320)	.10	-.05	-.27 (.003)	.09	-.15	.12 (.265)	.11	.06
**5**	.21 (<.001)	.05	.11	.10 (.075)	.06	.05	.20 (<.001)	.06	.10	-.17 (.002)	.06	-.08	-.19 (.001)	.06	-.09	-.07 (.209)	.06	-.04	-.02 (.793)	.06	-.01
**6**	.32 (<.001)	.01	.17	.41 (<.001)	.01	.21	.14 (<.001)	.02	.07	-.33 (<.001)	.01	-.17	-.30 (<.001)	.01	-.15	-.18 (<.001)	.01	-.09	-.06 (<.001)	.01	-.03
**7**	.23 (.001)	.07	.12	.20 (.005)	.07	.10	.19 (.008)	.07	.10	-.03 (.689)	.07	-.02	-.03 (.672)	.08	-.02	-.01 (859)	.07	-.01	-.18 (.018)	.08	-.09
**8**										-.12 (.009)	.05	-.05	.26 (<.001)	.05	.11	-.35 (<.001)	.05	-.15	.12 (.011)	.05	.05
**9**	.25 (.001)	.08	.11	.15 (.052)	.08	.07				-.10 (.194)	.08	-.05	-.25 (.001)	.07	-.11	.09 (.234)	.08	.04	-.02 (.772)	.08	-.01

Note. β = standardized regression beta; se = standard error; *r* = r-partial*;* Necessities is a composite of chores, caretaking, and/or family time. Overall leisure is a composite of active and passive leisure. Work is a composite or paid work and school work (Samples 8-9). Covariates in Sample 1 are: age, household income, employment status, marital status, and number of children. Covariates in Sample 2 are: age, employment status, marital status, number of children, and days since the survey was launched. Covariates in Sample 3 are: age, household income, employment status, marital status, and number of children. Covariates in Sample 4 are: age, household income, employment status, weekly work hours (apart from models with time-use work), household size, education level, number of children, and days since the survey was launched. Covariates in Sample 5-7 are: age, household income, household size, education level, number of children, and days since the survey was launched. Covariates in Sample 8 are: age and days since survey was launched. Covariates in Sample 9 are: age, race, socio-economic status (composite of parental education and income), household size, and days since the survey was launched.

Table S7b

Meta-Analysis of Gender (1=Female) on Time-Use During COVID-19 Across Samples

**Time-Use Composites**	**Included Samples**	**β**	**SE**	**CI Lower limit**	**CI Upper limit**	**PI Lower limit**	**PI Upper limit**	**z-value**	**Two-tailed p-value**
Necessities	3-7, 9	.29	.03	.21	.36	.15	.42	10.24	<.0001
Chores	3-7, 9	.18	.05	.05	.32	-.30	.67	3.55	<.0001
Caretaking + Caretaking /Family time	3-7	.22	.05	.09	.35	-.03	.46	4.70	<.0001
Caretaking	3-4	.35	.08	-.66	1.37	-.90	1.61	4.43	<.0001
Caretaking /Family time	5-7	.15	.01	.09	.21	.09	.21	10.48	<.0001
Overall leisure	1, 3-9	-.15	.04	-.25	-.04	-.55	.25	-3.28	.001
Active leisure	All samples	-.08	.07	-.24	.08	-.69	.53	-1.20	.229
Passive leisure	1, 3-9	-.11	.06	-.26	.04	-.45	.22	-1.86	.063
Work	All samples	-.04	.03	-.11	.04	-.20	.13	-1.14	.253

Note. CI = confidence interval. PI = prediction interval. Necessities is a composite of chores, caretaking, and/or family time. Overall leisure is a composite of active and passive leisure. Work is a composite or paid work and school work (Samples 8-9). Covariates in Sample 1 are: age, household income, employment status, marital status, and number of children. Covariates in Sample 2 are: age, employment status, marital status, number of children, and days since the survey was launched. Covariates in Sample 3 are: age, household income, employment status, weekly work hours, marital status, and number of children. Covariates in Sample 4 are: age, household income, employment status, household size, weekly work hours (apart from models with time-use work), education level, number of children, and days since the survey was launched. Covariates in Sample 5-7 are: age, household income, household size, education level, number of children, and days since the survey was launched. Covariates in Sample 8 are: age and days since survey was launched. Covariates in Sample 9 are: age, race, socio-economic status (composite of parental education and income), household size, and days since the survey was launched.

Fig. S1 | The meta-analytic effect of gender (1=women) on time-use samples based on models with covariates.

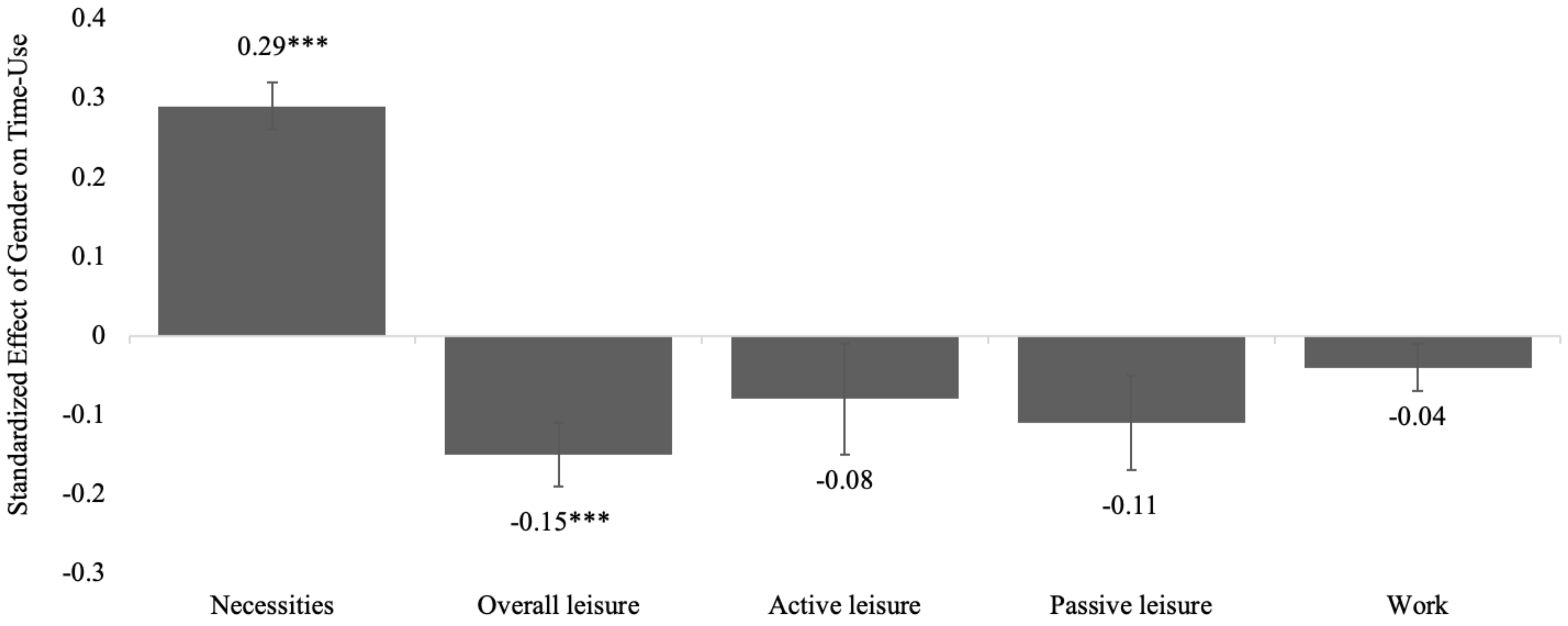

Note: For the necessities (composite) effect, we included Samples 3-7 and Sample 9. For the overall leisure effect, we included Sample 1 and Samples 3-9. For the active leisure effect, we included all samples. For the passive leisure effect, we included Sample 1 and Samples 4-9. While the meta-analytic effect for necessities and overall leisure was significant, it was only for necessities that the prediction interval did not include zero (see Table S17a for details and covariates included).

Individual and Meta-Analytic Effect Sizes of Gender Differences in Time-Use Across Samples (with Covariates)

Fig. S2a | Meta-Analysis: Gender Differences in Time Spent on Necessities (Composite) During COVID-19.

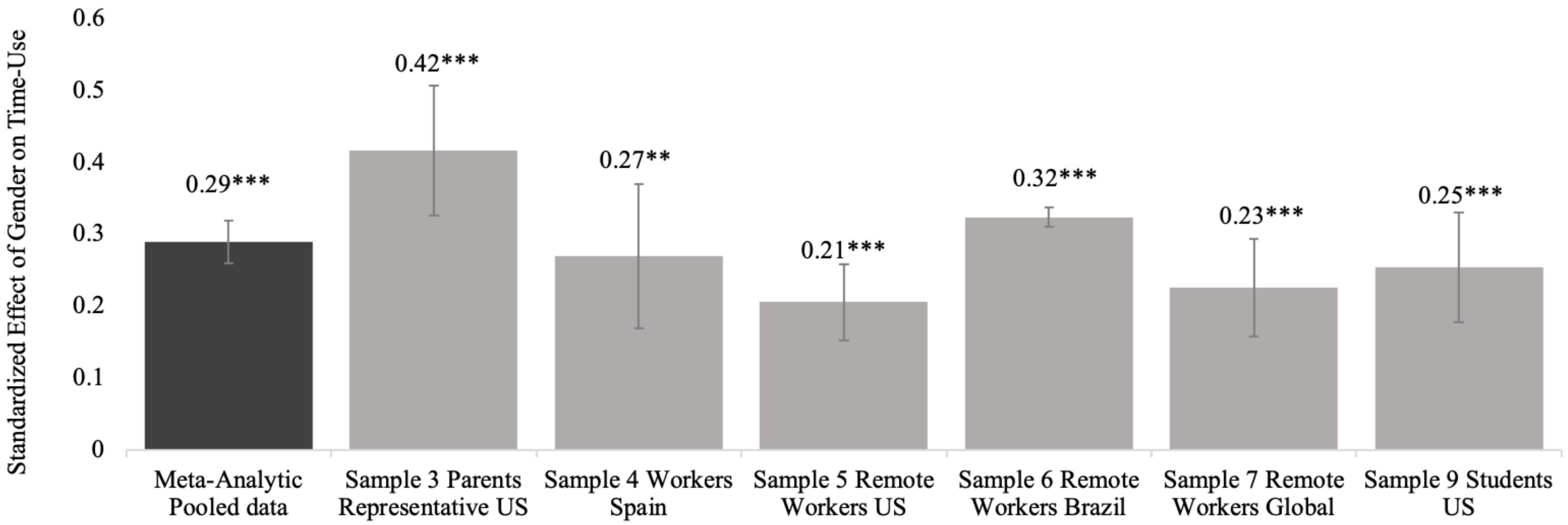

Note. Necessities is a composite of chores, caretaking, and/or family time. Covariates in Sample 1 are: age, household income, employment status, marital status, and number of children. Covariates in Sample 2 are: age, employment status, marital status, number of children, and days since the survey was launched. Covariates in Sample 3 are: age, household income, employment status, marital status, and number of children. Covariates in Sample 4 are: age, household income, employment status, household size, weekly work hours (apart from models with time-use work), education level, number of children, and days since the survey was launched. Covariates in Sample 5-7 are: age, household income, household size, education level, number of children, and days since the survey was launched. Covariates in Sample 8 are: age and days since survey was launched. Covariates in Sample 9 are: age, race, socio-economic status (composite of parental education and income), household size, and days since the survey was launched.

Fig. S2b | Meta-Analysis: Gender Differences in Time Spent on Chores During COVID-19.

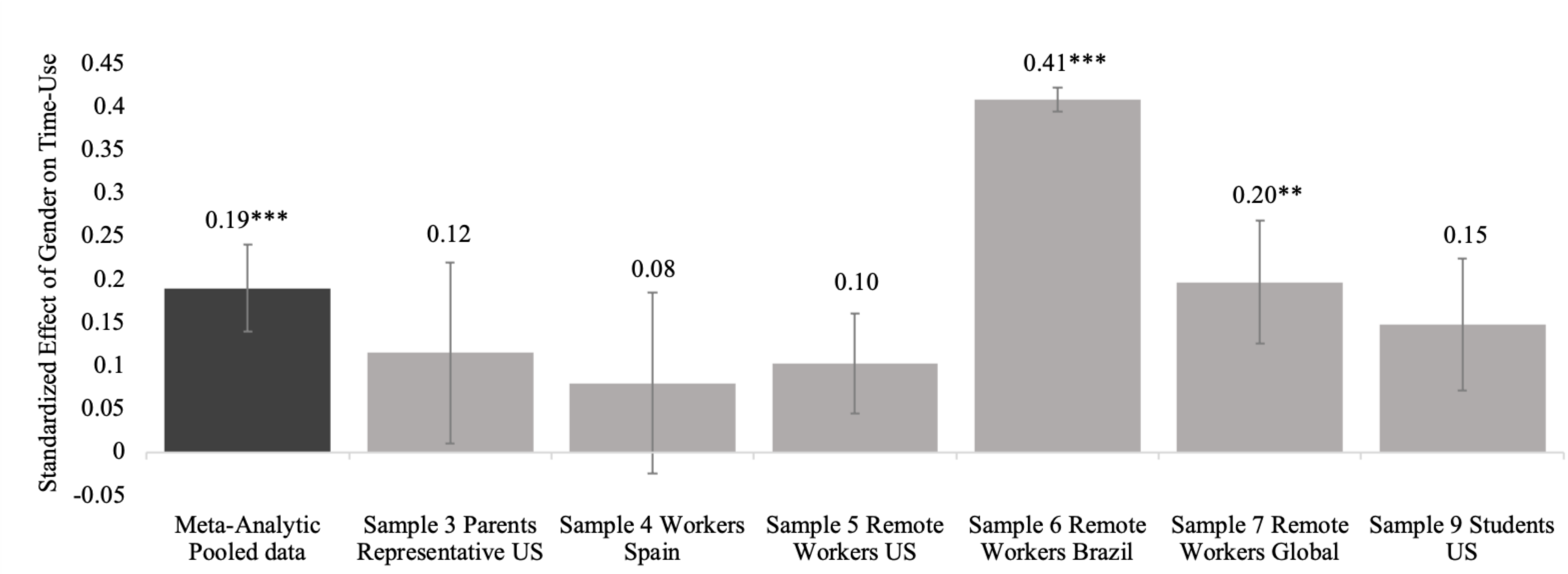

Note. Covariates in Sample 1 are: age, household income, employment status, marital status, and number of children. Covariates in Sample 2 are: age, employment status, marital status, number of children, and days since the survey was launched. Covariates in Sample 3 are: age, household income, employment status, marital status, and number of children. Covariates in Sample 4 are: age, household income, employment status, household size, education level, weekly work hours (apart from models with time-use work), number of children, and days since the survey was launched. Covariates in Sample 5-7 are: age, household income, household size, education level, number of children, and days since the survey was launched. Covariates in Sample 8 are: age and days since survey was launched. Covariates in Sample 9 are: age, race, socio-economic status (composite of parental education and income), household size, and days since the survey was launched.

Fig. S2c | Meta-Analysis: Gender Differences in Time Spent on Caretaking/Family Time (Samples 3-7) During COVID-19.

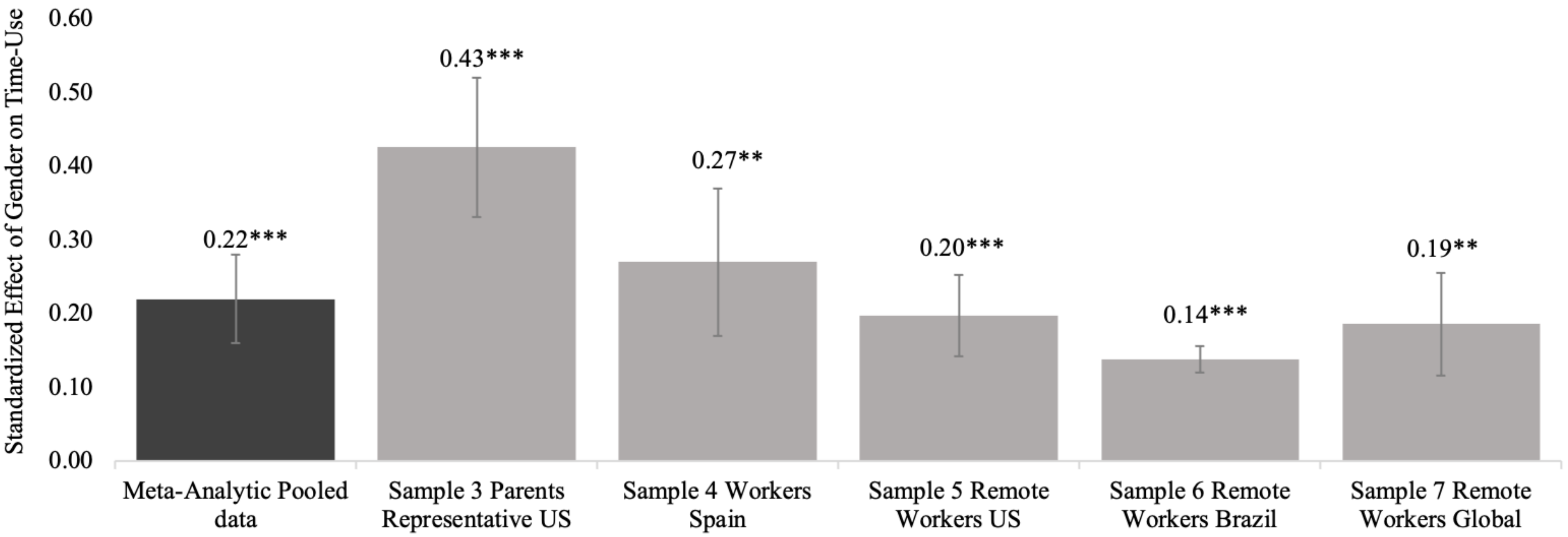

Note. Covariates in Sample 1 are: age, household income, employment status, marital status, and number of children. Covariates in Sample 2 are: age, employment status, marital status, number of children, and days since the survey was launched. Covariates in Sample 3 are: age, household income, employment status, marital status, and number of children. Covariates in Sample 4 are: age, household income, employment status, household size, weekly work hours (apart from models with time-use work), education level, number of children, and days since the survey was launched. Covariates in Sample 5-7 are: age, household income, household size, education level, number of children, and days since the survey was launched. Covariates in Sample 8 are: age and days since survey was launched. Covariates in Sample 9 are: age, race, socio-economic status (composite of parental education and income), household size, and days since the survey was launched.

Fig. S2d | Meta-Analysis: Gender Differences in Time Spent on Caretaking (Samples 3-4) During COVID-19.

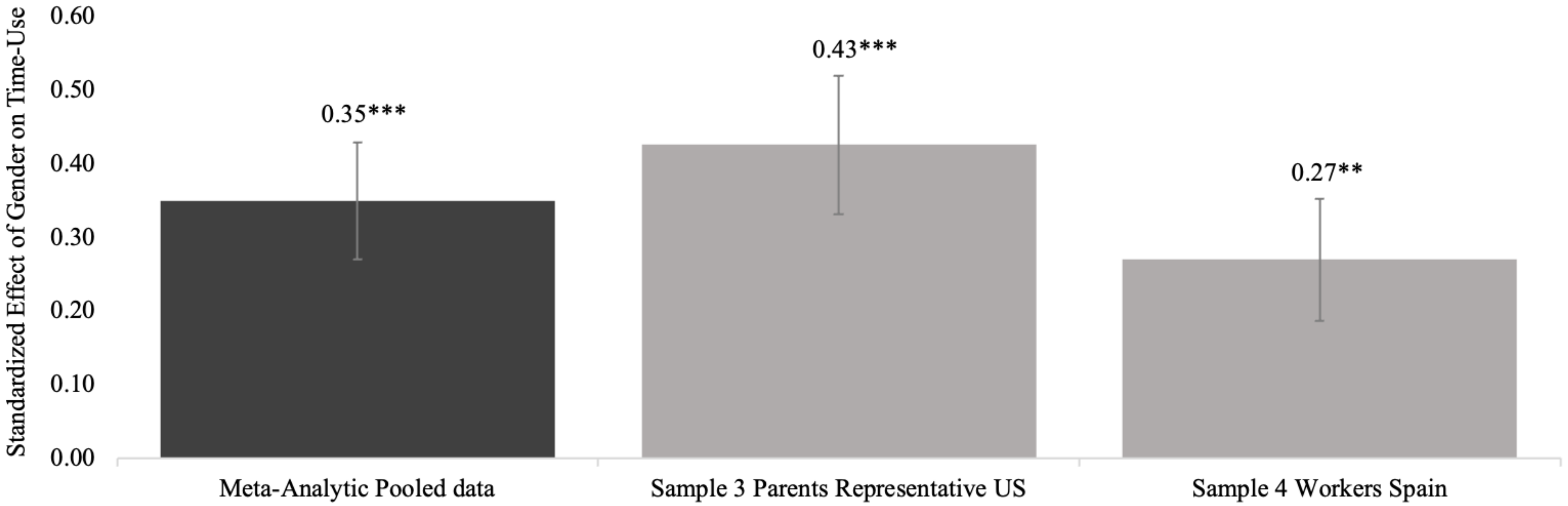

Note. Covariates in Sample 1 are: age, household income, employment status, marital status, and number of children. Covariates in Sample 2 are: age, employment status, marital status, number of children, and days since the survey was launched. Covariates in Sample 3 are: age, household income, employment status, marital status, and number of children. Covariates in Sample 4 are: age, household income, employment status, household size, education level, weekly work hours (apart from models with time-use work), number of children, and days since the survey was launched. Covariates in Sample 5-7 are: age, household income, household size, education level, number of children, and days since the survey was launched. Covariates in Sample 8 are: age and days since survey was launched. Covariates in Sample 9 are: age, race, socio-economic status (composite of parental education and income), household size, and days since the survey was launched.

Fig. S2e | Meta-Analysis: Gender Differences in Time Spent on Caretaking/Family Time (Samples 5-7) During COVID-19.

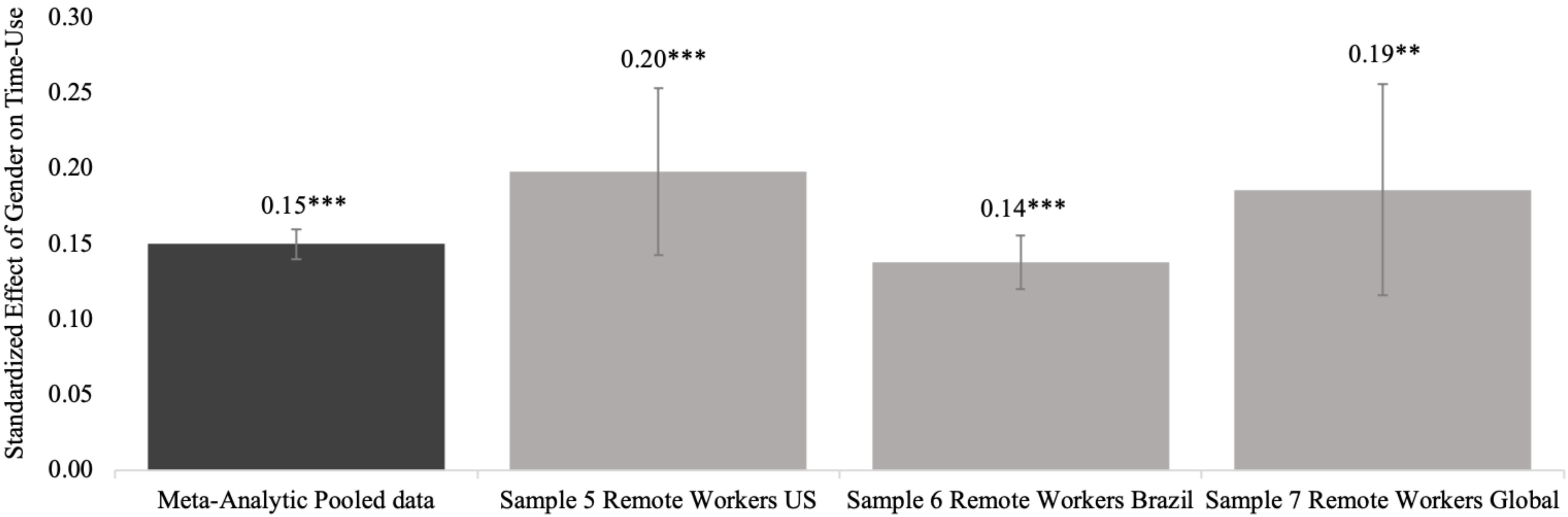

Note. Covariates in Sample 1 are: age, household income, employment status, marital status, and number of children. Covariates in Sample 2 are: age, employment status, marital status, number of children, and days since the survey was launched. Covariates in Sample 3 are: age, household income, employment status, marital status, and number of children. Covariates in Sample 4 are: age, household income, employment status, household size, education level, weekly work hours (apart from models with time-use work), number of children, and days since the survey was launched. Covariates in Sample 5-7 are: age, household income, household size, education level, number of children, and days since the survey was launched. Covariates in Sample 8 are: age and days since survey was launched. Covariates in Sample 9 are: age, race, socio-economic status (composite of parental education and income), household size, and days since the survey was launched.

Fig. S2f | Meta-Analysis: Gender Differences in Time Spent on Overall Leisure COVID-19.

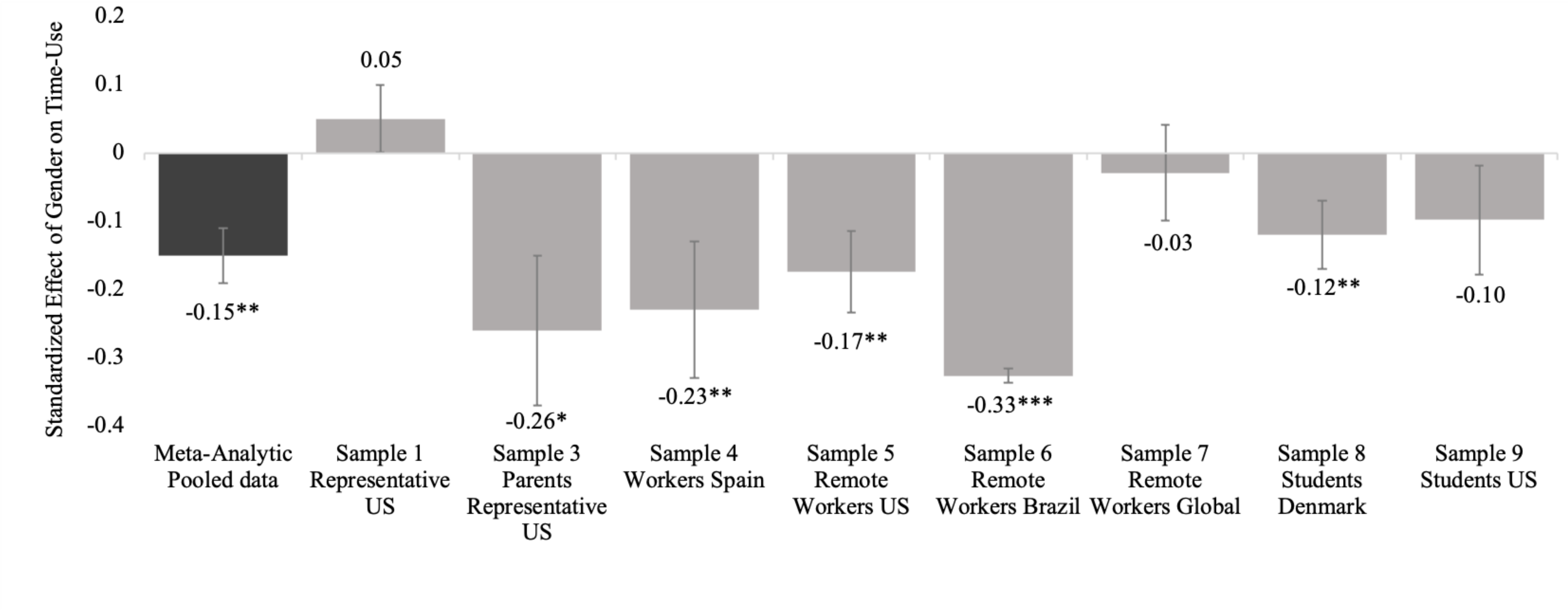

Note. Overall leisure is a composite of active and passive leisure. Covariates in Sample 1 are: age, household income, employment status, marital status, and number of children. Covariates in Sample 2 are: age, employment status, marital status, number of children, and days since the survey was launched. Covariates in Sample 3 are: age, household income, employment status, marital status, and number of children. Covariates in Sample 4 are: age, household income, employment status, household size, weekly work hours (apart from models with time-use work), education level, number of children, and days since the survey was launched. Covariates in Sample 5-7 are: age, household income, household size, education level, number of children, and days since the survey was launched. Covariates in Sample 8 are: age and days since survey was launched. Covariates in Sample 9 are: age, race, socio-economic status (composite of parental education and income), household size, and days since the survey was launched.

Fig. S2g | Meta-Analysis: Gender Differences in Time Spent on Active Leisure During COVID-19.

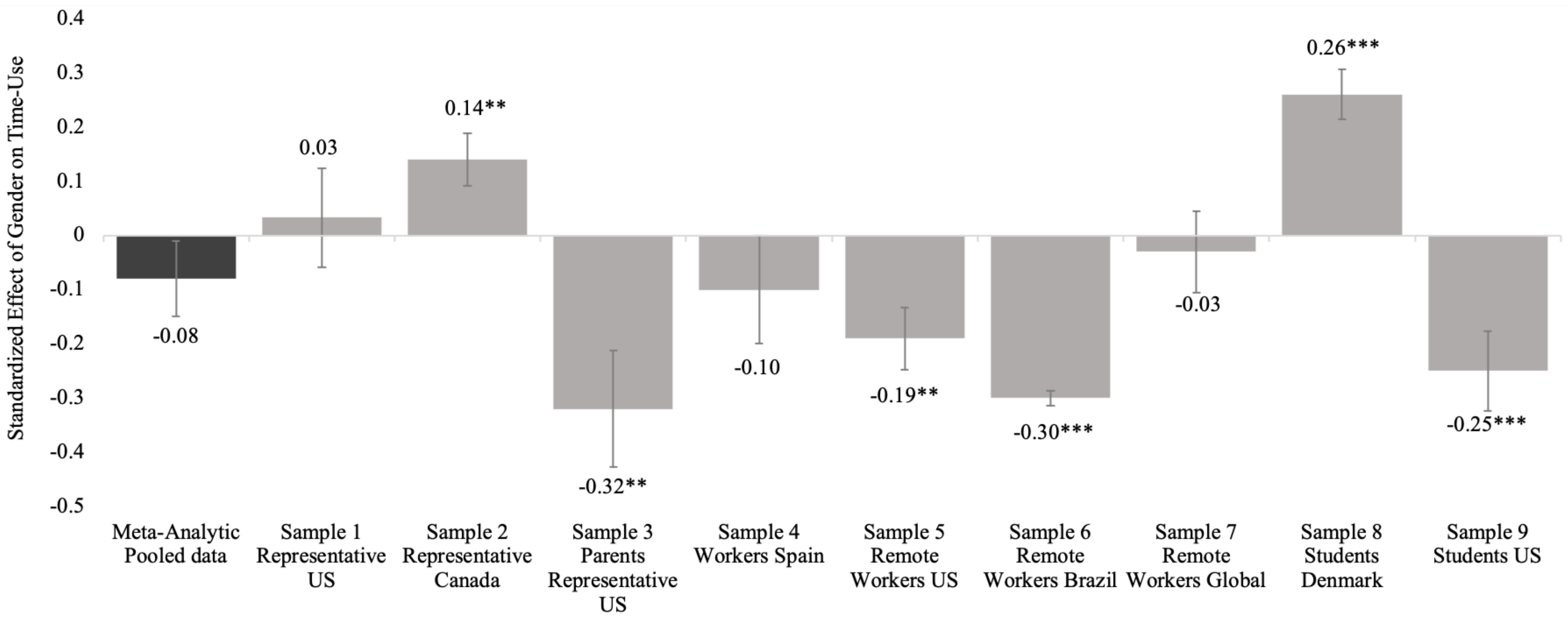

Note. Covariates in Sample 1 are: age, household income, employment status, marital status, and number of children. Covariates in Sample 2 are: age, employment status, marital status, number of children, and days since the survey was launched. Covariates in Sample 3 are: age, household income, employment status, marital status, and number of children. Covariates in Sample 4 are: age, household income, employment status, household size, weekly work hours (apart from models with time-use work), education level, number of children, and days since the survey was launched. Covariates in Sample 5-7 are: age, household income, household size, education level, number of children, and days since the survey was launched. Covariates in Sample 8 are: age and days since survey was launched. Covariates in Sample 9 are: age, race, socio-economic status (composite of parental education and income), household size, and days since the survey was launched.

Fig. S2h | Meta-Analysis: Gender Differences in Time Spent on Passive Leisure During COVID-19.

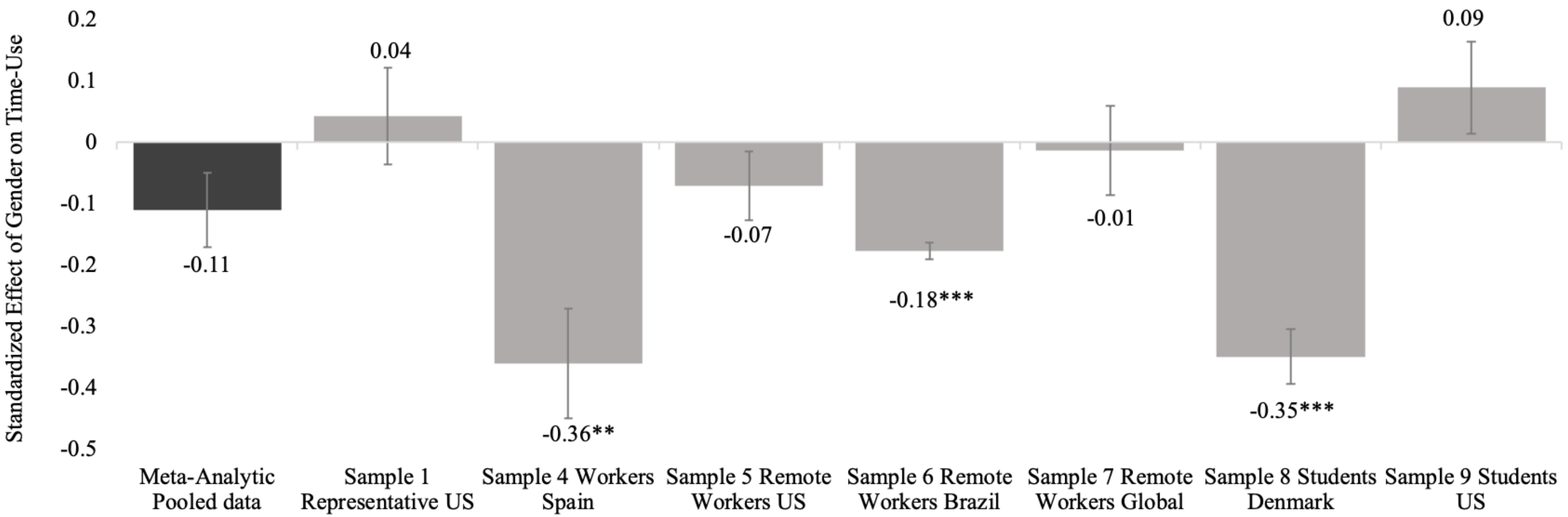

Note. Covariates in Sample 1 are: age, household income, employment status, marital status, and number of children. Covariates in Sample 2 are: age, employment status, marital status, number of children, and days since the survey was launched. Covariates in Sample 3 are: age, household income, employment status, marital status, and number of children. Covariates in Sample 4 are: age, household income, employment status, household size, education level, weekly work hours (apart from models with time-use work), number of children, and days since the survey was launched. Covariates in Sample 5-7 are: age, household income, household size, education level, number of children, and days since the survey was launched. Covariates in Sample 8 are: age and days since survey was launched. Covariates in Sample 9 are: age, race, socio-economic status (composite of parental education and income), household size, and days since the survey was launched.

Fig. S2i | Meta-Analysis: Gender Differences in Time Spent on Paid Work + School Work (Samples 8-9) During COVID-19,

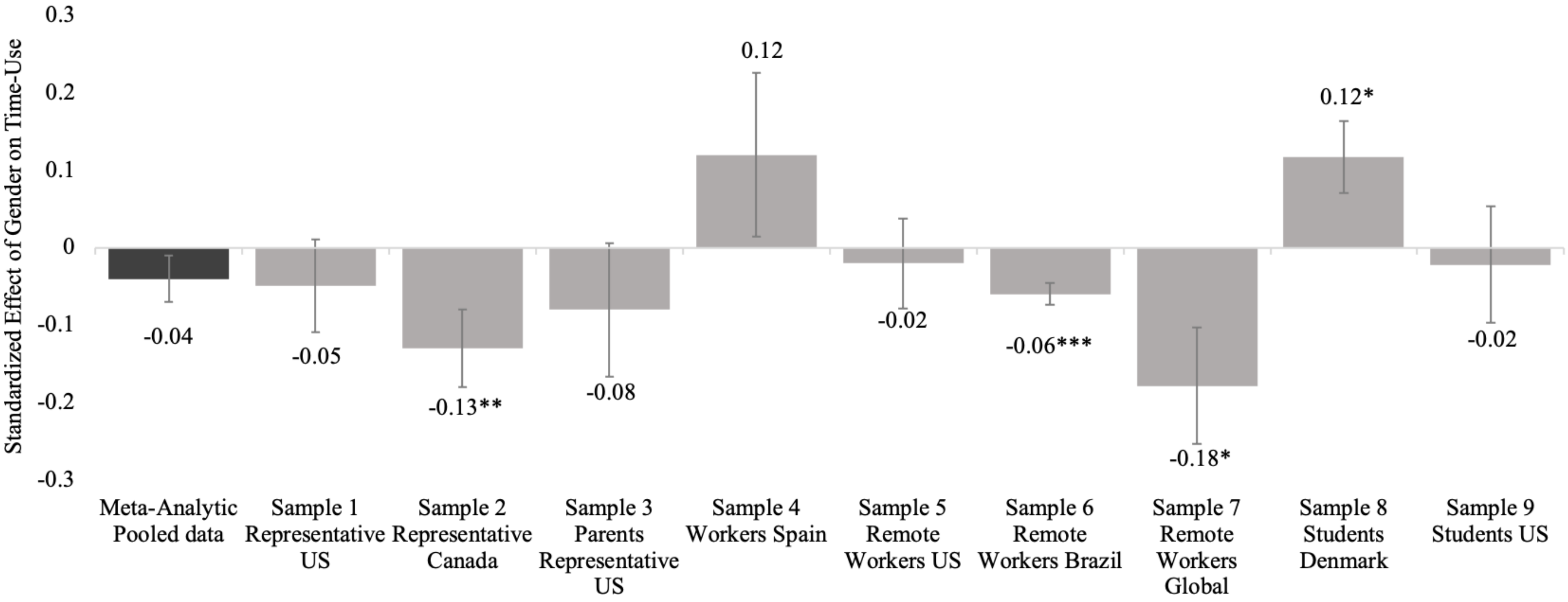

Note. Necessities is a composite of chores, caretaking, and/or family time. Covariates in Sample 1 are: age, household income, employment status, marital status, and number of children. Covariates in Sample 2 are: age, employment status, marital status, number of children, and days since the survey was launched. Covariates in Sample 3 are: age, household income, employment status, marital status, and number of children. Covariates in Sample 4 are: age, household income, employment status, household size, weekly work hours (apart from models with time-use work), education level, number of children, and days since the survey was launched. Covariates in Sample 5-7 are: age, household income, household size, education level, number of children, and days since the survey was launched. Covariates in Sample 8 are: age and days since survey was launched. Covariates in Sample 9 are: age, race, socio-economic status (composite of parental education and income), household size, and days since the survey was launched.

Meta-Analyses: Gender X Parental Status Differences in Time-Use Across Samples (with Covariates)

Table S8a

Standardized beta for gender (1=female) X parental status predicting time-use (with covariates)

	**Necessities**			**Chores**			**Caretaking/ Family time**			**Overall leisure**			**Active leisure**			**Passive leisure**			**Work**		
**Sample**	β (*p*-value)	(se)	r	β (*p*-value)	(se)	r	β (*p*-value)	(se)	r	β (*p*-value)	(se)	r	β (*p*-value)	(se)	r	β (*p*-value)	(se)	r	β (*p*-value)	(se)	r
**1**										-.20 (.098)	.12	-.08	-.15 (.416)	.18	-.04	-.12 (.465)	.16	-.04	.20 (.098)	.12	.08
**2**													.03 (.791)	.02	.01				-.02 (.844)	.10	-.01
**4**	-.32 (296)	.31	-.05	-.10 (.774)	.34	-.01	-.32 (.294)	.31	-.05	.47 (.101)	.29	.08	.09 (.765)	.29	.02	.67 (.014)	.27	.12	.28 (.400)	.33	.04
**5**	.56 <.001)	.11	.15	.18 (.128)	.12	.04	.52 (<.001)	.11	.14	-.33 (.003)	.11	-.08	-.18 (.124)	.12	-.04	-.28 (.018)	.12	-.07	-.19 (.101)	.12	-.05
**6**	.25 <.001)	.03	.07	.18 (<.001)	.03	.05	.21 (<.001)	.03	.06	-.14 (<.001)	.03	-.04	-.02 (.483)	.03	-.01	-.15 (<.001)	.03	-.04	-.12 (<.001)	.03	-.03
**7**	.28 (.032)	.13	.08	.43 (.002)	.14	.11	.29 (.021)	.14	.08	.02 (.861)	.14	.006	.03 (.835)	.14	.01	.02 (.870)	.14	.01	-.20 (.179)	.15	-.05

Note. *β* = standardized regression beta; se = standard error; *r* = r-partial. Necessities is a composite of chores, caretaking, and/or family time. Overall leisure is a composite of active and passive leisure. Work is a composite or paid work and school work (Samples 8-9). Covariates in Sample 1 are: age, household income, employment status, marital status, and number of children. Covariates in Sample 2 are: age, employment status, marital status, number of children, and days since the survey was launched. Covariates in Sample 3 are: age, household income, employment status, marital status, and number of children. Covariates in Sample 4 are: age, household income, employment status, weekly work hours (apart from models with time-use work), household size, education level, number of children, and days since the survey was launched. Covariates in Sample 5-7 are: age, household income, household size, education level, number of children, and days since the survey was launched. Covariates in Sample 8 are: age and days since survey was launched. Covariates in Sample 9 are: age, race, socio-economic status (composite of parental education and income), household size, and days since the survey was launched.

Table S8b

Meta-Analysis of the Gender X Parental Status Interaction Term on Time-Use During COVID-19

**Time-Use Composites**	**Included samples**	**β**	**SE**	**CI Lower limit**	**CI Upper limit**	**PI Lower limit**	**PI Upper limit**	**z-value**	**p-value**
Necessities	4-7	.29	.13	-.14	.72	-.40	.98	2.17	.030
Chores	4-7	.21	.05	.04	.37	-.03	.45	3.91	<.0001
Caretaking + Caretaking /Family time	4-7	.27	.13	-.15	.68	-.42	.95	2.04	.041
Caretaking /Family time	5-7	.32	.09	-.08	.73	-.44	1.09	3.44	<.0001
Overall leisure	1, 4-7	-.13	.09	-.38	.12	-.53	.28	-1.41	.159
Active leisure	1-2, 4-7	-.02	.02	-.08	.03	-.08	.03	-1.27	.204
Passive leisure	1, 4-7	-.07	.12	-.41	.28	-.62	.49	-.55	.581
Work	1-2, 4-7	-.06	.06	-.22	.10	-.35	.23	-.96	.339

Note. CI = confidence interval. PI = prediction interval. Given that caretaking among parents and non-parents was included only in Sample 4, we could not run a meta-analysis on this individual item as we did with gender differences in time-use Necessities is a composite of chores, caretaking, and/or family time. Overall leisure is a composite of active and passive leisure. Work is a composite or paid work and school work (Samples 8-9). Covariates in Sample 1 are: age, household income, employment status, marital status, and number of children. Covariates in Sample 2 are: age, employment status, marital status, number of children, and days since the survey was launched. Covariates in Sample 3 are: age, household income, employment status, marital status, and number of children. Covariates in Sample 4 are: age, household income, employment status, household size, weekly work hours (apart from models with time-use work), education level, number of children, and days since the survey was launched. Covariates in Sample 5-7 are: age, household income, household size, education level, number of children, and days since the survey was launched. Covariates in Sample 8 are: age and days since survey was launched. Covariates in Sample 9 are: age, race, socio-economic status (composite of parental education and income), household size, and days since the survey was launched..

Table S8c

Sub-Group Meta-Analysis of Gender Among Parents vs. Non-Parents on Time-Use During COVID-19

		NON-PARENTS						PARENTS					
	**Included samples**	**β**	**se**	**CI**	**PI**	**z**	**p**	**β**	**se**	**CI**	**PI**	**z**	**p**
Necessities	4-7	.15	.08	[-.10, .39]	[-.31, .60]	1.90	.058	.44	.06	[25, .63]	[.13, .75]	7.27	<.001
Chores	4-7	.16	.08	[-.09,.40]	[-.47, .78]	2.05	.040	.34	.07	[.09,.42]	[-.15, .66]	3.71	<.001
Caretaking/ Family time	5-7	.05	.01	[-.01,.10]	[-.01, .10]	3.78	<.001	.36	.07	[.04, .67]	[-.24, .95]	4.92	<.001
Caretaking + Caretaking/ Family time	4-7	.04	.04	[-.08, .17]	[-.14, .23]	1.10	.271	.33	.06	[.14, .51]	[-.02, .68]	5.62	<.001
Overall leisure	1, 4-7	-.11	.07	[-.42,.20]	[-.71, .49]	-.94	.349	-.22	.08	[-.46, .03]	[-.81, .38]	-2.64	.008
Active leisure	1-2, 4-7	-.08	.07	[-.26, .09]	[-.60, .43]	-1.21	.226	-.12	.07	[-.31, .07]	[-.67, .43]	-1.5	.134
Passive leisure	1, 4-7	-.06	.06	[-.39.26]	[-.57, .44]	-.54	.587	-.15	.06	[-.32,.02]	[-.51, .21]	-2.45	.014
Work	1-2, 4-7	-.03	.03	[-.09,.04]	[-.12, .07]	-1.08	.279	-.08	.05	[-.21,.05]	[-.32, .15]	-1.65	.100

Note. CI = confidence interval. PI = prediction interval. Necessities is a composite of chores, caretaking, and/or family time. Overall leisure is a composite of active and passive leisure. Work is a composite or paid work. Covariates in Sample 1 are: age, household income, employment status, marital status, and number of children. Covariates in Sample 2 are: age, employment status, marital status, number of children, and days since the survey was launched. Covariates in Sample 4 are: age, household income, employment status, weekly work hours (apart from models with time-use work), household size, education level, number of children, and days since the survey was launched. Covariates in Sample 5-7 are: age, household income, household size, education level, number of children, and days since the survey was launched. Covariates in Sample 8 are: age and days since survey was launched. Covariates in Sample 9 are: age, race, socio-economic status (composite of parental education and income), household size, and days since the survey was launched.

Fig. S3a | The meta-analytic effect of gender (1=women) and gender by parental status (1=yes) based on models with covariates.

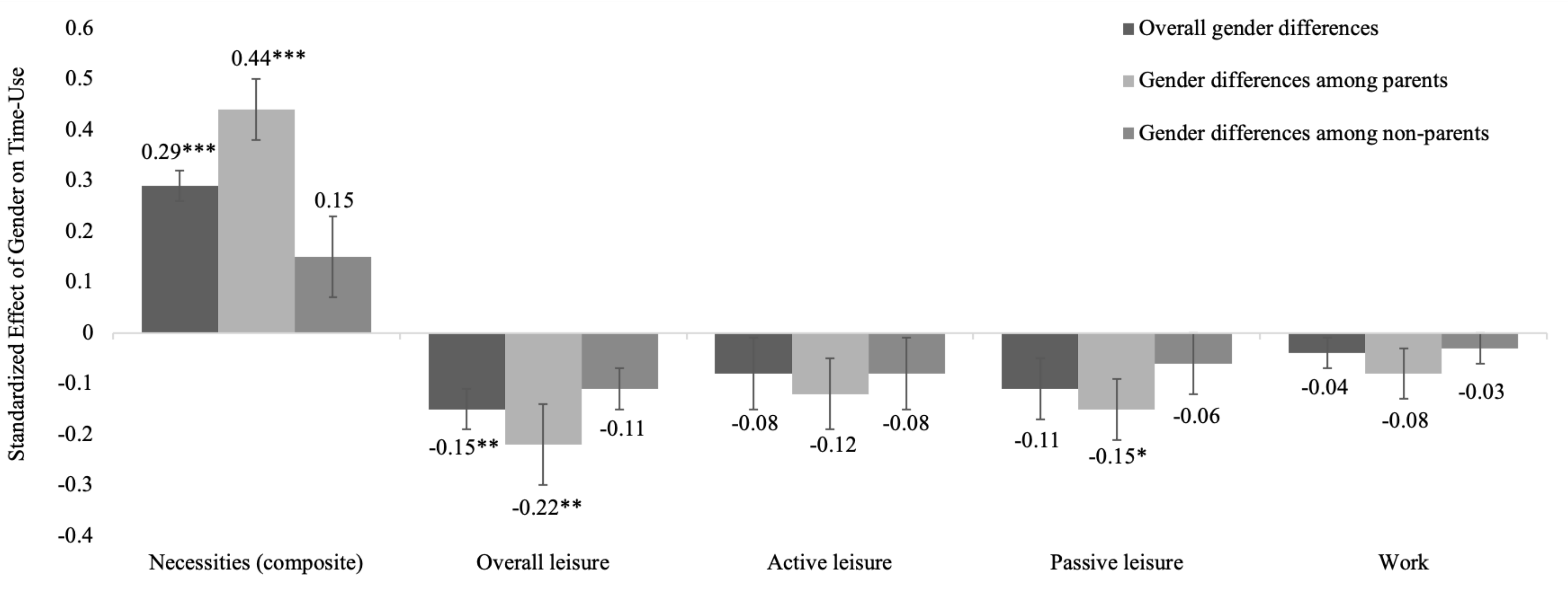

Note. For the necessities (composite) effect, we included Samples 4-7. For the overall leisure effect, we included Sample 1 and 4-8. For the active leisure effect, we included Samples 1-2, 4-7. For the passive leisure effect, we included Sample 1 and 4-7. For the work effect, we included Samples 1-2, 4-7. The most robust meta-analytic effect emerged for necessities among parents as indicated by the confidence and prediction interval that did not cross zero (see Table S19c for details and covariates included). Covariates in Sample 1 are: age, household income, employment status, marital status, and number of children. Covariates in Sample 2 are: age, employment status, marital status, number of children, and days since the survey was launched. Covariates in Sample 3 are: age, household income, employment status, marital status, and number of children. Covariates in Sample 4 are: age, household income, employment status, household size, weekly work hours (apart from models with time-use work), education level, number of children, and days since the survey was launched. Covariates in Sample 5-7 are: age, household income, household size, education level, number of children, and days since the survey was launched. Covariates in Sample 8 are: age and days since survey was launched. Covariates in Sample 9 are: age, race, socio-economic status (composite of parental education and income), household size, and days since the survey was launched.

Fig. S4 | The meta-analytic effect of gender (1=women) and of gender by parental status (1=yes) based on models with covariates.

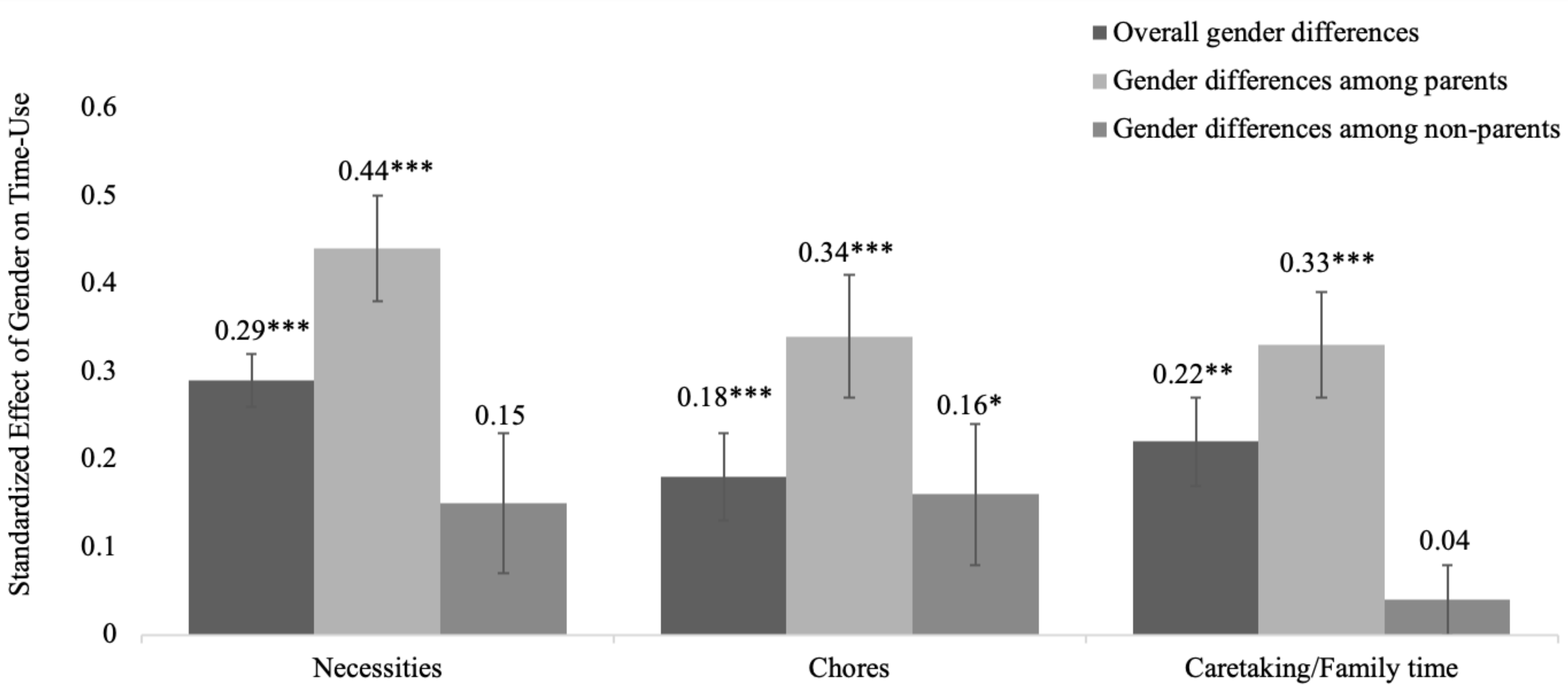

Note. Covariates in Sample 1 are: age, household income, employment status, marital status, and number of children. Covariates in Sample 2 are: age, employment status, marital status, number of children, and days since the survey was launched. Covariates in Sample 3 are: age, household income, employment status, marital status, and number of children. Covariates in Sample 4 are: age, household income, employment status, household size, weekly work hours (apart from models with time-use work), education level, number of children, and days since the survey was launched. Covariates in Sample 5-7 are: age, household income, household size, education level, number of children, and days since the survey was launched. Covariates in Sample 8 are: age and days since survey was launched. Covariates in Sample 9 are: age, race, socio-economic status (composite of parental education and income), household size, and days since the survey was launched.

Meta-Analyses: Gender and Gender X Parental Status Differences in Happiness Across Samples (with Covariates)

Table S9a

Standardized beta and r-partial for gender (1=female) predicting happiness (with covariates)

	Gender main effects			Gendr X Parental Status		
**Sample**	**β (p-value)**	**(se)**	**r**	**β (p-value)**	**(se)**	**r**
**1**	-.20 (.034)	.10	-.10	.17 (.375)	.19	.04
**2**	.01 (.860)	.07	.01	.05 (.370)	.14	.01
**3**	-.02 (.856)	.11	-.01	--	--	--
**4**	-.05 (.632)	.10	-.02	-.30 (.309)	.29	-.05
**5**	.05 (.412)	.06	.02	-.01 (.926)	.12	-.003
**6**	.01 (.513)	.01	.05	-.04 (.188)	.03	-.01
**7**	.05 (.510)	.07	.02	-.25 (.093)	.15	-.06
**8**	.10 (.026)	.05	.04	--	--	--
**9**	-.27 (<.001)	.08	-.12	--	--	--

Note. β = standardized regression beta; se = standard error; r = r-partial. Covariates in Sample 1 are: age, household income, employment status, marital status, and number of children. Covariates in Sample 2 are: age, employment status, marital status, number of children, and days since the survey was launched. Covariates in Sample 3 are: age, household income, employment status, marital status, and number of children. Covariates in Sample 4 are: age, household income, employment status, household size, education level, weekly work hours (apart from models with time-use work), number of children, and days since the survey was launched. Covariates in Sample 5-7 are: age, household income, household size, education level, number of children, and days since the survey was launched. Covariates in Sample 8 are: age and days since survey was launched. Covariates in Sample 9 are: age, race, socio-economic status (composite of parental education and income), household size, and days since the survey was launched.

Table S9b
Meta-Analysis of the Gender and Gender X Parental Status on Happiness During COVID-19

**Time-Use Composites**	**Included samples**	**β**	**SE**	**CI Lower limit**	**CI Upper limit**	**PI Lower limit**	**PI Upper limit**	**z-value**	**p-value**
Gender	1-9	-.02	.04	-.11	.07	-.20	.17	-.42	.673
Gender X Parental Status	1-2, 4-7	-.04	.02	-.10	.03	-.10	.03	-1.53	.127
**Sub-group moderation analysis**									
Non-Parents	1-2, 4-7	.02	.05	-.10	.14	-.17	.21	.047	.640
Parents	1-2, 4-7	-.02	.02	-.06	.02	-.06	.02	-1.01	.312
Non-Parents	1-7	.02	.02	-.10	.14	-.17	.21	1.62	.105
Parents	1-7	-.02	.01	-.05	.02	-.05	.02	-1.13	.257

Note. CI = confidence interval. PI = prediction interval. Covariates in Sample 1 are: age, household income, employment status, marital status, and number of children. Covariates in Sample 2 are: age, employment status, marital status, number of children, and days since the survey was launched. Covariates in Sample 3 are: age, household income, employment status, marital status, and number of children. Covariates in Sample 4 are: age, household income, employment status, weekly work hours (apart from models with time-use work), household size, education level, number of children, and days since the survey was launched. Covariates in Sample 5-7 are: age, household income, household size, education level, number of children, and days since the survey was launched. Covariates in Sample 8 are: age and days since survey was launched. Covariates in Sample 9 are: age, race, socio-economic status (composite of parental education and income), household size, and days since the survey was launched.

Fig. S5 | Gender differences in happiness.

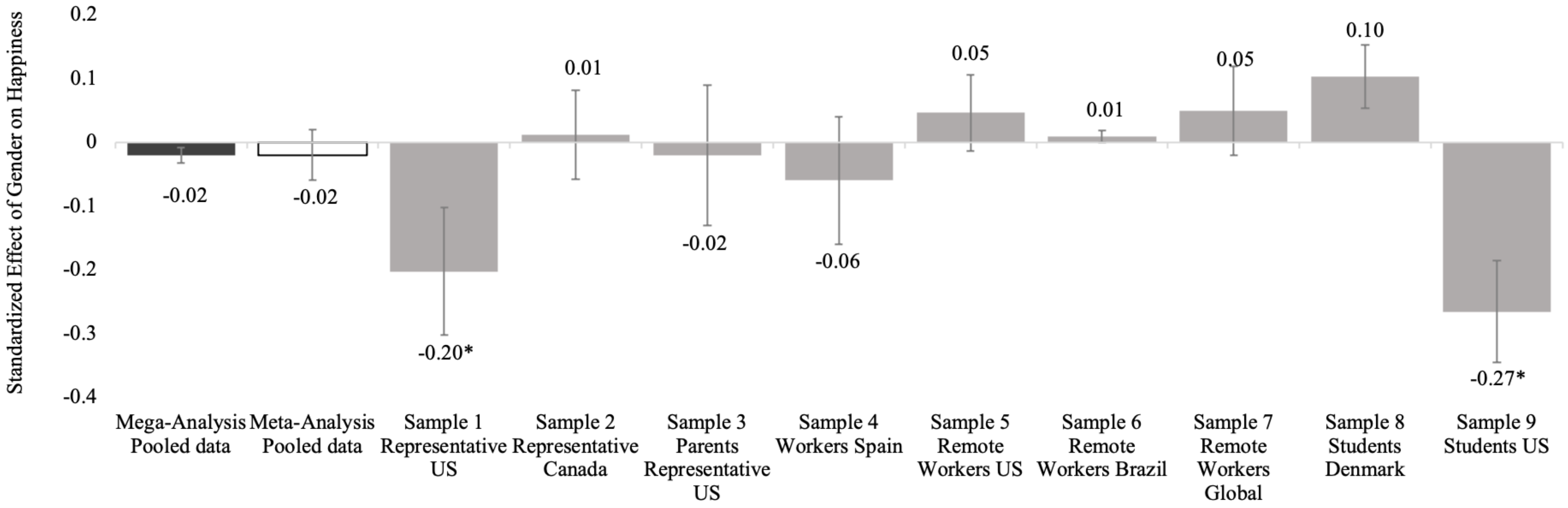

Note. The mega-analyses were conducted without covariates. The meta-analyses and the sample-specific effects are based on models with covariates as follows. Covariates in Sample 1 are: age, household income, employment status, marital status, and number of children. Covariates in Sample 2 are: age, employment status, marital status, number of children, and days since the survey was launched. Covariates in Sample 3 are: age, household income, employment status, marital status, and number of children. Covariates in Sample 4 are: age, household income, employment status, household size, weekly work hours (apart from models with time-use work), education level, number of children, and days since the survey was launched. Covariates in Sample 5-7 are: age, household income, household size, education level, number of children, and days since the survey was launched. Covariates in Sample 8 are: age and days since survey was launched. Covariates in Sample 9 are: age, race, socio-economic status (composite of parental education and income), household size, and days since the survey was launched.

Meta-Analyses: Time-Use and Happiness Across Samples (with Covariates)

Table S10a

Standardized beta for time-use predicting overall happiness (with covariates)

	**Necessities→ Happiness**			**Chores→ Happiness**			**Caretaking/ Family time→ Happiness**			**Overall leisure→ Happiness**			**Active leisure→ Happiness**			**Passive leisure→ Happiness**			**Work→ Happiness**		
**Sample**	β (*p*-value)	(se)	r	β (*p*-value)	(se)	r	β (*p*-value)	(se)	r	β (*p*-value)	(se)	r	β (*p*-value)	(se)	r	β (*p*-value)	(se)	r	β (*p*-value)	(se)	r
**1**	β (*p*-value)	(se)	r	β (*p*-value)	(se)	r	β (*p*-value)	(se)	r	β (*p*-value)	(se)	r	β (*p*-value)	(se)	r	β (*p*-value)	(se)	r	β (*p*-value)	(se)	r
**2**										-.12 (.128)	.08	-.07	.01 (.038)	.00	.10	-001 (.002)	.00	-.15	.00 (.128)	.00	.07
**3**																			-.05 (.407)	.06	-.03
**4**	.002 (.968)	.06	-.002	-.03 (.598)	.05	-.03	.02 (.770)	.06	.02	.13 (.013)	.05	.13	.04 (.437)	.06	.03				-.06 (.367)	.06	-.05
**5**	-.05 (.467)	.06	-.04	-.21 (.667)	.05	-.21	-.04 (.566)	.06	-.04	.16 (.002)	.05	.15	.17 (.001)	.05	.17	-.04 (.417)	.05	-.04	-.10 (.051)	.05	-.10
**6**	-.04 (.186)	.03	-.04	-.09 (.002)	.03	-.09	.00 (.931)	.03	.00	-.07 (.021)	.03	-.06	.23 (<.001)	.05	.22	-.15 (<.001)	.03	-.15	.02 (.051)	.03	.02
**7**	-.02 (.003)	.01	-.02	-.10 (<.001)	.01	-.10	.03 (<.001)	.01	.03	.01 (.067)	.01	.01	.08 (.002)	.03	.09	-.07 (<.001)	.01	-.07	-.04 (.388)	.01	-.04
**8**	.05 (.224)	.04	.04	-.03 (.418)	.04	-.03	.09 (.02)	.04	.09	-.03 (.383)	.04	-.03	.10 (<.001)	.01	.10	-.09 (<.001)	.04	-.10	-.04 (<.001)	.04	-.04
**9**										-.20 (.313)	.02	-.02	.07 (<.001)	.04	.07	-.17 (<.001)	.02	-.17	.02 (.316)	.02	.02

Note. *β* = standardized regression beta; se = standard error; *r* = r-partial. Necessities is a composite of chores, caretaking, and/or family time. Overall leisure is a composite of active and passive leisure. Work is a composite or paid work and school work (Samples 8-9). Covariates in Sample 1 are: age, household income, employment status, marital status, and number of children. Covariates in Sample 2 are: age, employment status, marital status, number of children, and days since the survey was launched. Covariates in Sample 3 are: age, household income, employment status, marital status, and number of children. Covariates in Sample 4 are: age, household income, employment status, weekly work hours (apart from models with time-use work), household size, education level, number of children, and days since the survey was launched. Covariates in Sample 5-7 are: age, household income, household size, education level, number of children, and days since the survey was launched. Covariates in Sample 8 are: age and days since survey was launched. Covariates in Sample 9 are: age, race, socio-economic status (composite of parental education and income), household size, and days since the survey was launched. See Meta-Analysis files on OSF for additional details and sample-specific analyses.

Table S10b

Meta-Analysis of Time-Use on Overall Happiness (with covariates)

**Time-Use Composites**	**Included samples**	**β**	**SE**	**CI Lower limit**	**CI Upper limit**	**PI Lower limit**	**PI Upper limit**	**z-value**	**p-value**
Necessities	3-7, 9	-.02	.01	-.04	.01	-.04	.01	-1.94	.026
Chores	3-7, 9	-.08	.03	-.15	-.01	-.21	.06	-2.81	.005
Caretaking + Caretaking /Family time	3-7	.03	.01	-.01	.07	-.03	.08	1.88	.060
Caretaking /Family time	3-4	-.01	.03	-.39	.37	-.39	.37	-.33	.739
Caretaking	5-7	.3	.02	-.06	.12	-.10	.16	1.53	.125
Overall leisure	1, 3-9	-.01	.04	-.11	.1	-.29	.28	-.16	.875
Active leisure	1-9	.12	.03	.06	.19	-.08	.33	4.44	<.001
Passive leisure	1, 4-9	-.09	.02	-.14	-.04	-.26	.08	-4.07	<.001
Work	1-9	-.01	.01	-.04	.02	-.08	.05	-1.01	.312

Note. CI = confidence interval. PI = prediction interval. Necessities is a composite of chores, caretaking, and/or family time. Overall leisure is a composite of active and passive leisure. Work is a composite or paid work and school work (Samples 8-9). Covariates in Sample 1 are: age, household income, employment status, marital status, and number of children. Covariates in Sample 2 are: age, employment status, marital status, number of children, and days since the survey was launched. Covariates in Sample 3 are: age, household income, employment status, marital status, and number of children. Covariates in Sample 4 are: age, household income, employment status, household size, weekly work hours (apart from models with time-use work), education level, number of children, and days since the survey was launched. Covariates in Sample 5-7 are: age, household income, household size, education level, number of children, and days since the survey was launched. Covariates in Sample 8 are: age and days since survey was launched. Covariates in Sample 9 are: age, race, socio-economic status (composite of parental education and income), household size, and days since the survey was launched.

Mega-Analyses Results

Table S11a. Comparing Mega-Analysis Models with a Random Intercept vs. Random Slope Using Likelihood Ratio Tests

DV	IV	*chi2*	*p*-value
Happiness	Gender	4.80	0.0907
Neccesities (composite)	Gender	0.0651	0.0033
Chores	Gender	52.83	0.000
Caretaking/Family Time	Gender	23.81	0.000
Overall leisure	Gender	90.42	0.000
Active Leisure	Gender	146.99	0.000
Passive Leisure	Gender	36.42	0.000
Work	Gender	14.41	0.001
Happiness	Neccesities (composite)	3.22	0.073
Happiness	Overall leisure	22.04	0.000
Happiness	Work	10.74	0.001
Happiness	Chores	196.91	0.000
Happiness	Caretaking/Family Time	10.86	0.001
Happiness	Active Leisure	39.31	0.000
Happiness	Passive Leisure	24.92	0.000

*Note.*We tested the null hypothesis that there is no significant difference between the models i) a random intercept model of the IV predicting DV and ii) a random intercept and slope model of the IV predicting DV. The rejection of the null (*p*-value < .05) suggests that adding the random slope to the random intercept model improves the fit of the model. Unstructured covariances were used in all random slope models except for the model with active leisure where an independent covariance structure was used due to the convergence issues which are common with unstructured covariances (Boedhoe et al., 2019).

Table S11b. Mega-Analysis Findings for Differences in Happiness and Time Use by Gender (Without Covariates)

	Effect Size	95% CI	Two-tailed p-value
**Happiness**	-0.02	[-0.041,0.004]	0.103
**Neccesities**	0.28	[0.135,0.419]	0.000
**Overall Leisure**	-0.08	[-0.187,0.030]	0.158
**Work**	-0.08	[-0.170,0.015]	0.100
Necessities			
**Chores**	0.24	[0.141,0.344]	0.000
**Caretaking/Family Time**	0.22	[0.020,0.411]	0.031
Leisure			
**Active Leisure**	-0.05	[-0.168,0.071]	0.429
**Passive Leisure**	-0.09	[-0.222,0.049]	0.213

Note. CI= confidence interval. Beta estimates represent coefficients in standard deviation units. Pooled data from 9 samples were used in the analysis. Necessities, chores and caretaking were not measured in Samples 1, 2 and 8. Passive leisure was not measured in Samples 2 and 3. Overall leisure is a composite of active and passive leisure. Work is paid work except for Samples 8-9 where it is paid work and schoolwork. Caretaking/family time was measured as 'taking care of family (kids or elderly) in Samples 3 and 4, and 'taking care of others or spending time with family' in Samples 5, 6, and 7.Random slope models were used in mega-analysis of pooled data for necessities, overall leisure, active leisure, passive leisure, chores, caretaking/family time and work. Covariances were unstructured (except for active leisure where an independent covariance was specified).The main effects for necessities remain robust after applying Bonferroni corrections, that is, using an alpha/5 in evaluating the statistical significance of 5 time use models that we preregistered (necessities, overall leisure, active leisure, passive leisure, work).

Table S11c.
Mega-Analysis of Differences in Happiness and Time Use by Gender X Parental Status (Without Covariates)

	Estimate for Gender X Parents Interaction	95% CI	Two-tailed p-value	Estimate for Gender (Parents = 1)	95% CI	p-value	Estimate for Gender (Parents = 0)	95% CI	Two-tailed p-value
**Happiness**	-0.030	[-0.083,0.015]	0.180	-0.04	[-0.078,-0.004]	0.030	-0.01	[-0.040,0.025]	0.652
**Necessities**	0.25	[0.199,0.294]	0.000	0.48	[0.365,0.597]	0.000	0.23	[0.119,0.349]	0.000
**Total Leisure**	-0.13	[-0.174, -0.077]	0.000	-0.15	[-0.292, 0.001]	0.052	-0.02	[-0.168,0.128]	0.791
**Work**	-0.10	[-0.152,-0.053]	0.000	-0.18	[-0.269,-0.083]	0.000	-0.07	[-0.168,0.020]	0.125
Necessities									
**Chores**	0.17	[0.117,0.216]	0.000	0.33	[0.204,0.463]	0.000	0.17	[0.036,0.298]	0.012
**Caretaking/Family Time**	0.20	[0.150,0.249]	0.000	0.34	[0.181,0.509]	0.000	0.15	[-0.020,0.310]	0.085
Leisure									
**Active Leisure**	-0.03	[-0.079,0.020]	0.245	-0.11	[-0.223, 0.004]	0.060	-0.08	[-0.195,0.035]	0.172
**Passive Leisure**	-0.14	[-0.192,-0.093]	0.000	-0.12	[-0.247,-0.003]	0.044	0.02	[-0.105,0.139]	0.781

Note. CI= confidence interval. Beta estimates represent coefficients in standard deviation units. Pooled data from 9 samples were used in the analysis. Necessities, chores and caretaking were not measured in Samples 1, 2 and 8. Passive leisure was not measured in Samples 2 and 3. Overall leisure is a composite of active and passive leisure. Work is paid work except for Samples 8-9 where it is paid work and schoolwork. Caretaking/family time was measured as 'taking care of family (kids or elderly) in Samples 3 and 4, and 'taking care of others or spending time with family' in Samples 5, 6, and 7. Random slope models were used in mega-analysis of pooled data for necessities, overall leisure, active leisure, passive leisure, chores, caretaking/family time and work. Covariances were unstructured (except for active leisure where an independent covariance was specified). Estimates for parents=1 or parents=0 were retrieved from the interaction model using the postestimation commands (marginsplot) in Stata 15.1.

Table S11d.
Mega-Analysis Findings for the Relationship Between Happiness and Time Use (Without Covariates)

	Effect Size	95% CI	Two-tailed p-value
**Neccesities**	0.00	[-.003, .003]	0.925
**Overall Leisure**	-0.05	[-.054, -.042]	0.000
**Work**	0.01	[.004, .008]	0.000
Necessities			
**Chores**	-0.03	[-.043, -.025]	0.000
**Caretaking/Family Time**	-0.07	[-.082, -.060]	0.000
**Caretaking**	0.00	[-.002, .007]	0.218
Leisure			
**Active Leisure**	0.01	[.005, .013]	0.000
**Passive Leisure**	-0.04	[-.045, -.030]	0.000

Note. CI= confidence interval. Beta estimates represent coefficients in standard deviation units. Pooled data from 9 samples were used in the analysis. Necessities, chores and caretaking were not measured in Samples 1, 2 and 8. Passive leisure was not measured in Samples 2 and 3. Overall leisure is a composite of active and passive leisure. Work is paid work except for Samples 8-9 where it is paid work and schoolwork. Caretaking/family time was measured as 'taking care of family (kids or elderly) in Samples 3 and 4, and 'taking care of others or spending time with family' in Samples 5, 6, and 7. The caretaking analysis was restricted to Sample 3 to capture the pure measure of taking care of family. Guided by ikelihood ratio tests, random slopes were used in all models and random intercept model was used when necessities was a predictor.. Covariances were unstructured (except for active leisure where an independent covariance was specified). The main effects for chores remain robust after applying Bonferroni corrections, which involve using an alpha/5 for testing 5 time use models that we preregistered (necessities, overall leisure, active leisure, passive leisure, work) in evaluating the statistical significance.

Table S11e.
Indirect Effects from Gender to Happiness via Time Use in Mega-Analysis

		Without Covariates			*With Covariates*		
**Full Sample**	**Mediator**	**Estimate**	**95% CI**	**Two-tailed p-value**	**Estimate**	**95% CI**	**Two-tailed p-value**
Single mediator	**Necessities**	0.00	[-.003, .003]	0.925	0.00	[-.005, .003]	0.548
Multiple mediators	**Chores**	-0.05	[-.054, -.042]	0.000	-0.05	[-.057, -.045]	0.000
	**Caretaking/Family Time**	0.01	[.004, .008]	0.000	0.01	[.004, .009]	0.000
**Among Parents**							
Single mediator	**Necessities**	-0.03	[-.043, -.025]	0.000	-0.03	[-.040, -.024]	0.000
Multiple mediators	**Chores**	-0.07	[-.082, -.060]	0.000	-0.07	[-.082, -.061]	0.000
	**Caretaking/Family Time**	0.00	[-.002, .007]	0.218	0.00	[-.000, .006]	0.147
**Among Non-Parents**							
Single mediator	**Necessities**	0.01	[.005, .013]	0.000	0.01	[.004, .012]	0.000
Multiple mediators	**Chores**	-0.04	[-.045, -.030]	0.000	-0.04	[-.049, -.034]	0.000
	**Caretaking/Family Time**	0.00	[-.004,.005]	0.812	0.00	[-.002, .006]	0.231

*Note.*CI= confidence interval. Beta estimates represent coefficients in standard deviation units. Pooled data from 6 samples were used in the analysis (necessities, chores and caretaking were not measured in Samples 1, 2 and 8). Generalized structural equation modeling with random intercepts were used. Indirect effects were calculated by using the nlcom command on Stata 15.1.

Table S11f.

Differences in Happiness and Time Use by Gender (Without Covariates)

	Happiness			*Necessities*			*Chores*			*Caretaking/Family Time*		
	**beta**	**95% CI**	**p-value**	**beta**	**95% CI**	**p-value**	**beta**	**95% CI**	**p-value**	**beta**	**95% CI**	**p-value**
Pooled data	-0.02	[-0.041,0.004]	0.103	0.28	[0.135,0.419]	0.000	0.24	[0.141,0.344]	0.000	0.22	[0.020,0.411]	0.031
Sample 1	-0.2	[-0.386,-0.011]	0.038	NA	NA	NA	NA	NA	NA	NA	NA	NA
Sample 2	-0.06	[-0.203,0.078]	0.383	NA	NA	NA	NA	NA	NA	NA	NA	NA
Sample 3	-0.15	[-0.346,0.047]	0.136	0.73	[0.545,0.912]	0.000	0.27	[0.080,0.470]	0.006	0.70	[0.517,0.886]	0.000
Sample 4	-0.04	[-0.189,0.101]	0.551	0.21	[0.049,0.371]	0.011	0.13	[-0.035,0.286]	0.126	0.18	[0.019,0.341]	0.029
Sample 5	-0.01	[-0.112,0.098]	0.898	0.12	[0.012,0.223]	0.029	0.13	[0.028,0.241]	0.014	0.09	[-0.018,0.196]	0.105
Sample 6	-0.02	[-0.046,0.007]	0.148	0.27	[0.247,0.300]	0.000	0.44	[0.413,0.465]	0.000	0.05	[0.028,0.082]	0.000
Sample 7	0.04	[-0.094,0.166]	0.587	0.24	[0.106,0.367]	0.000	0.27	[0.143,0.405]	0.000	0.14	[0.005,0.269]	0.042
Sample 8	0.1	[0.022,0.185]	0.013	NA	NA	NA	NA	NA	NA	NA	NA	NA
Sample 9	-0.26	[-0.409,-0.117]	0.000	0.25	[0.099,0.392]	0.001	0.17	[0.025,0.318]	0.021	NA	NA	NA

Note. Beta estimates represent coefficients in standard deviation units. Random slope models were used in mega-analysis of pooled data for necessities, chores and taking care of others. Covariances were unstructured. Random intercept model was used for the mega-analysis of happiness. Linear regression analysis was used in sample-specific analaysis. Taking Care of Others V1 was measured by a question that assessed 'taking care of others' in Samples 3 and 4, and a question that assessed 'taking care of others or spending time with family' in Samples 5, 6, and 7.

Table S11g.
Differences in Happiness and Time Use by Gender (Without Covariates)

	Total Leisure			*Active Leisure*			*Passive Leisure*			*Work*		
	**beta**	**95% CI**	**p-value**	**beta**	**95% CI**	**p-value**	**beta**	**95% CI**	**p-value**	**beta**	**95% CI**	**p-value**
Pooled data	-0.24	[-0.262,-0.216]	0.000	-0.05	[-0.168,0.071]	0.429	-0.09	[-0.222,0.049]	0.213	-0.08	[-0.170,0.015]	0.100
Sample 1	0.22	[0.035,0.411]	0.020	0.13	[-0.063,0.314]	0.193	0.17	[-0.020,0.357]	0.08	-0.22	[-0.411,-0.035]	0.020
Sample 2	0.16	[0.010,0.302]	0.036	0.16	[0.010,0.302]	0.036	NA	NA	NA	-0.15	[-0.295,-0.003]	0.045
Sample 3	-0.36	[-0.553,-0.165]	0.000	-0.36	[-0.553,-0.165]	0.000	NA	NA	NA	-0.41	[-0.599,-0.213]	0.000
Sample 4	-0.15	[-0.307,0.014]	0.073	0.09	[-0.074,0.249]	0.286	-0.35	[-0.511,-0.194]	0.000	0.09	[-0.074,0.248]	0.291
Sample 5	-0.08	[-0.182,0.029]	0.158	-0.12	[-0.225,-0.016]	0.024	0.000	[-0.102,0.110]	0.942	0.000	[-0.101,0.109]	0.940
Sample 6	-0.3	[-0.329,-0.276]	0.000	-0.29	[-0.314,-0.261]	0.000	-0.15	[-0.182,-0.128]	0.000	-0.04	[-0.070,-0.017]	0.001
Sample 7	0.03	[-0.102,0.159]	0.668	-0.02	[-0.148,0.112]	0.787	0.05	[-0.078,0.185]	0.426	-0.2	[-0.328,-0.066]	0.003
Sample 8	-0.12	[-0.208,-0.034]	0.007	0.25	[0.158,0.332]	0.000	-0.35	[-0.431,-0.259]	0.000	0.12	[0.031,0.205]	0.008
Sample 9	-0.11	[-0.260,0.036]	0.136	-0.26	[-0.406,-0.111]	0.001	0.08	[-0.067,0.228]	0.286	-0.02	[-0.173,0.124]	0.744

Note. Beta estimates represent coefficients in standard deviation units. Random slope models were used in mega-analysis of pooled data for total leisure, active leisure, passive leisure and work. Covariance was unstructured for total leisure, passive leisure, and work; and independent for active leisure. Linear regression analysis was used in sample-specific analaysis. Taking Care of Others V1 was measured by a question that assessed 'taking care of others' in Samples 3 and 4, and a question that assessed 'taking care of others or spending time with family' in Samples 5, 6, and 7.

Table S11h.
Differences in Happiness and Time Use by Gender (Without Covariate)

	Taking Care of Family			*Caretaking/Family Time*		
	**beta**	**95% CI**	**p-value**	**beta**	**95% CI**	**p-value**
Pooled data	NA	NA	NA	NA	NA	NA
Sample 1	NA	NA	NA	NA	NA	NA
Sample 2	NA	NA	NA	NA	NA	NA
Sample 3	0.7	[0.517,0.886]	0.000	NA	NA	NA
Sample 4	0.18	[0.019,0.341]	0.029	NA	NA	NA
Sample 5	NA	NA	NA	0.09	[-0.018,0.196]	0.105
Sample 6	NA	NA	NA	0.05	[0.028,0.082]	0.000
Sample 7	NA	NA	NA	0.14	[0.005,0.269]	0.042
Sample 8	NA	NA	NA	NA	NA	NA
Sample 9	NA	NA	NA	NA	NA	NA

Note. Beta estimates represent coefficients in standard deviation units. Linear regression analysis was used in sample-specific analaysis. Taking Care of Others V1 was measured by a question that assessed 'taking care of others' in Samples 3 and 4, and a question that assessed 'taking care of others or spending time with family' in Samples 5, 6, and 7.

Table S11i.
Mega-Analysis Findings for the Relationship Between Happiness and Time Use (Without Covariates)

	Effect Size	95% CI	Two-tailed p-value
**Neccesities**	0.00	[-0.013,0.011]	0.876
**Overall Leisure**	0.01	[-0.041,0.066]	0.639
**Work**	0.00	[-0.031,0.023]	0.755
Necessities			
**Chores**	-0.06	[-0.098,-0.017]	0.006
**Caretaking/Family Time**	0.04	[-0.036,0.111]	0.315
**Caretaking**	-0.08	[-.174, .021]	0.126
Leisure			
**Active Leisure**	0.12	[0.067,0.177]	0.000
**Passive Leisure**	-0.11	[-0.151,-0.079]	0.000

Note. CI= confidence interval. Beta estimates represent coefficients in standard deviation units. Pooled data from 9 samples were used in the analysis. Necessities, chores and caretaking were not measured in Samples 1, 2 and 8. Passive leisure was not measured in Samples 2 and 3. Overall leisure is a composite of active and passive leisure. Work is paid work except for Samples 8-9 where it is paid work and schoolwork. Caretaking/family time was measured as 'taking care of family (kids or elderly) in Samples 3 and 4, and 'taking care of others or spending time with family' in Samples 5, 6, and 7. The caretaking analysis was restricted to Sample 3 to capture the pure measure of taking care of family. Guided by ikelihood ratio tests, random slopes were used in all models and random intercept model was used when necessities was a predictor.. Covariances were unstructured (except for active leisure where an independent covariance was specified). The main effects for chores remain robust after applying Bonferroni corrections, which involve using an alpha/5 for testing 5 time use models that we preregistered (necessities, overall leisure, active leisure, passive leisure, work) in evaluating the statistical significance.

Sample 9 (924): All Results for Longitudinal Sample

Table S12a

Demographic characteristics of survey respondents in longitudinal student data

n	924
Mean (*SD*) age, years	21 (1.6)
% Female	73%
% White	62%
% Black	3%
% Asian	21%
% Hispanic	7%
% Other Race	7%
Mean (SD) parents' education, years	5.71 (1.16)
Median parents' income	8.50 (3.84)
Mean (SD) no. cohabitants	3.03 (1.72)
Mean (*SD*) no. days in survey, years	12.48 (8.62)

Note. Age ranged from 18 to 25. Education was measured as the average of 7 educational levels (from some grade school to post-grad degree) for parents 1 and 2. The mean and median of the income was equal. Range for no of cohabitants was 0 to 10 and for no of days since survey launch was 0 to 44.

Table S12b
Descriptive statistics for the main study variables

	Time 1		Time 2	
	**Mean**	**SD**	**Mean**	**SD**
Subjective well-being				
Overall Happiness	5.63	1.92	6.08	1.87
Positive Affect	3.25	0.75	3.39	0.77
Negative Affect	3.08	0.84	2.88	0.85
Meaning in Life	4.13	1.54	4.16	1.45
Time-Use Composites				
% Work	0.17	0.12	0.18	0.13
% Overall leisure	0.4	0.14	0.39	0.15
% Active leisure	0.17	0.1	0.17	0.1
% Passive leisure	0.23	0.13	0.22	0.13
% Necessities	0.1	0.06	0.1	0.06

Note. Global happiness was measured with the question: "Taking all things together, how happy would you say you are?" rated from 0 (Not at all) to 10 (Extremely). Positive affect was measured with three positive feelings experienced last week, and negative affect with three negative feelings experienced last week (1 = Very rarely, 5 = Very often/always). Work was captured with 3-items (work, study, commuting). Overall leisure was the sum of active and passive leisure. Active leisure was the sum of time spent praying/worshipping/meditating, socializing, exercising, intimate relations, going outdoors, hobbies. Passive leisure was the sum of time spent watching tv, napping/resting, relaxing, doing nothing. Necessities was measured as the sum of time spent shopping, personal hygiene, preparing food, doing housework. For each composite, an episode weighted statistics was computed where the amount of time that respondents reported spending on each activity is weighted by the total amount of time spent in all measured activities: work, active leisure, passive leisure, necessities and other measured activities (phone/computer use, eating, waiting +other, social media use).

Table S12c

Regression analyses predicting time-use differences at Time 1 by socio-demographic status (Sample 9)

	**Model 1 Work**		**Model 2 Overall leisure**		**Model 3 Active Leisure**		**Model 4 Passive Leisure**		**Model 5 Necessities**	
	β	*p-value*	*β*	*p-value*	*β*	*p-value*	*β*	*p-value*	*β*	*p-value*
Gender (1 = female)	.00	.826	-.01	.215	-.02	.001	.01	.257	.14	.002
SES	.00	.362	.00	.578	.01	.001	-.02	.002	-.04	.114
Education	.00	.584	.01	.446	.01	.116	-.01	.049	.00	.962
Income	.00	.656	.00	.922	.01	.128	-.01	.218	-.02	.410

Note. Work was captured with 3-items (work, study, commuting). Overall leisure was the sum of active and passive leisure. Active leisure was the sum of time spent praying/worshipping/meditating, socializing, exercising, intimate relations, going outdoors, hobbies. Passive leisure was the sum of time spent watching tv, napping/resting, relaxing, doing nothing. Necessities was measured as the sum of time spent shopping, personal hygiene, preparing food, doing housework For each composite, an episode weighted statistics was computed where the amount of time that respondents reported spending on each activity is weighted by the total amount of time spent in all measured activities: work, active leisure, passive leisure, necessities and other measured activities (phone/computer use, eating, waiting +other, social media use). The weighted time-use necessities were log transformed as skewness was greater than 3. Covariates were: age (range = 18 to 25), gender (female=1, male=0), race (dummy variables for being White, Black, Asian, Hispanic and Other), SES (education and income were entered as a composite or individually depending on the model), number of days since survey launch, number of co-habitants (range = 0 to 10).

Table S12d

Regression analyses predicting differences in SWB at Time 1 by socio-demographic status (Sample 9)

	**Model 1 SWB**		**Model 2 Overall happiness**		**Model 3 Positive Affect**		**Model 4 Negative Affect**		**Model 5 Meaning in Life**	
	β	*p-value*	*β*	*p-value*	*β*	*p-value*	*β*	*p-value*	*β*	*p-value*
Gender (1 = female)	-.23	0	-.25	.001	-.15	.052	.29	.00	-.24	.001
SES	.1	.006	.1	.017	.15	0	-.05	.204	.07	.081
Education	.01	.865	0	.980	.04	.409	.02	.717	0	.977
Income	.07	.052	.06	.134	.11	.013	-.05	.269	.06	.177

Note. Work was captured with 3-items (work, study, commuting). Overall leisure was the sum of active and passive leisure. Active leisure was the sum of time spent praying/worshipping/meditating, socializing, exercising, intimate relations, going outdoors, hobbies. Passive leisure was the sum of time spent watching tv, napping/resting, relaxing, doing nothing. Necessities was measured as the sum of time spent shopping, personal hygiene, preparing food, doing housework For each composite, an episode weighted statistics was computed where the amount of time that respondents reported spending on each activity is weighted by the total amount of time spent in all measured activities: work, active leisure, passive leisure, necessities and other measured activities (phone/computer use, eating, waiting +other, social media use). The weighted time-use necessities were log transformed as skewness was greater than 3. Covariates were: age (range = 18 to 25), gender (female=1, male=0), race (dummy variables for being White, Black, Asian, Hispanic and Other), SES (education and income were entered as a composite or individually depending on the model), number of days since survey launch, number of co-habitants (range = 0 to 10).

Table S12e

Regression analyses predicting differences in SWB at Time 2 by socio-demographic status (Sample 9)

	**Model 1 SWB**		**Model 2 Overall happiness**		**Model 3 Positive Affect**		**Model 4 Negative Affect**		**Model 5 Meaning in Life**	
	β	*p-value*	*β*	*p-value*	*β*	*p-value*	*β*	*p-value*	*β*	*p-value*
Gender (1 = female)	-.16	.013	-.17	.026	-.05	.529	.28	.00	-.16	.034
SES	.06	.082	.09	.024	.1	.019	0	.986	.10	.021
Education	.00	.988	.03	.517	.01	.791	.04	.38	.04	.436
Income	.05	.168	.05	.221	.08	.076	-.03	.516	.06	.147

Note. Work was captured with 3-items (work, study, commuting). Overall leisure was the sum of active and passive leisure. Active leisure was the sum of time spent praying/worshipping/meditating, socializing, exercising, intimate relations, going outdoors, hobbies. Passive leisure was the sum of time spent watching tv, napping/resting, relaxing, doing nothing. Necessities was measured as the sum of time spent shopping, personal hygiene, preparing food, doing housework For each composite, an episode weighted statistics was computed where the amount of time that respondents reported spending on each activity is weighted by the total amount of time spent in all measured activities: work, active leisure, passive leisure, necessities and other measured activities (phone/computer use, eating, waiting +other, social media use). The weighted time-use necessities were log transformed as skewness was greater than 3. Covariates were: age (range = 18 to 25), gender (female=1, male=0), race (dummy variables for being White, Black, Asian, Hispanic and Other), SES (education and income were entered as a composite or individually depending on the model), number of days since survey launch, number of co-habitants (range = 0 to 10).

Table S12f

Regression analyses predicting differences in SWB at Time 2 by socio-demographic status - controlling for SWB at Time 1 (Sample 9)

	**Model 1 SWB**		**Model 2 Overall happiness**		**Model 3 Positive Affect**		**Model 4 Negative Affect**		**Model 5 Meaning in Life**	
	β	*p-value*	*β*	*p-value*	*β*	*p-value*	*β*	*p-value*	*β*	*p-value*
Gender (1 = female)	-.01	.832	-.01	.827	.04	.524	.12	.053	-.01	.915
SES	.00	.924	.03	.303	.01	.740	.03	.415	.05	.126

Note. Work was captured with 3-items (work, study, commuting). Overall leisure was the sum of active and passive leisure. Active leisure was the sum of time spent praying/worshipping/meditating, socializing, exercising, intimate relations, going outdoors, hobbies. Passive leisure was the sum of time spent watching tv, napping/resting, relaxing, doing nothing. Necessities was measured as the sum of time spent shopping, personal hygiene, preparing food, doing housework For each composite, an episode weighted statistics was computed where the amount of time that respondents reported spending on each activity is weighted by the total amount of time spent in all measured activities: work, active leisure, passive leisure, necessities and other measured activities (phone/computer use, eating, waiting +other, social media use). The weighted time-use necessities were log transformed as skewness was greater than 3. Covariates were: age (range = 18 to 25), gender (female=1, male=0), race (dummy variables for being White, Black, Asian, Hispanic and Other), SES (education and income were entered as a composite or individually depending on the model), number of days since survey launch, number of co-habitants (range = 0 to 10).

Table S12g
Regression analyses predicting SWB at Time 2 by time-use composites at Time 1 (Sample 9)

	**Model 1 SWB**		**Model 2 Overall happiness**		**Model 3 Positive Affect**		**Model 4 Negative Affect**		**Model 5 Meaning in Life**	
	β	*p-value*	*β*	*p-value*	*β*	*p-value*	*β*	*p-value*	*β*	*p-value*
% Work	.33	.171	.43	.113	.08	.764	-.47	.08	.19	.499
% Overall leisure	.34	.098	.18	.446	.39	.09	-.45	.052	.38	.104
% Active leisure	1.81	.000	2.05	.000	1.83	.000	-1.56	.000	1.74	.000
% Passive leisure	-.67	.002	-1.00	.000	-.63	.011	.40	.106	-.58	.021
% Necessities	.08	.121	.13	.016	.08	.154	-.01	.795	.04	.501

Note. Work was captured with 3-items (work, study, commuting). Overall leisure was the sum of active and passive leisure. Active leisure was the sum of time spent praying/worshipping/meditating, socializing, exercising, intimate relations, going outdoors, hobbies. Passive leisure was the sum of time spent watching tv, napping/resting, relaxing, doing nothing. Necessities was measured as the sum of time spent shopping, personal hygiene, preparing food, doing housework For each composite, an episode weighted statistics was computed where the amount of time that respondents reported spending on each activity is weighted by the total amount of time spent in all measured activities: work, active leisure, passive leisure, necessities and other measured activities (phone/computer use, eating, waiting +other, social media use). The weighted time-use necessities were log transformed as skewness was greater than 3. Covariates were: age (range = 18 to 25), gender (female=1, male=0), race (dummy variables for being White, Black, Asian, Hispanic and Other), SES (education and income were entered as a composite or individually depending on the model), number of days since survey launch, number of co-habitants (range = 0 to 10).

Table S12h

Regression analyses predicting SWB at Time 2 by time-use composites at Time 1 - controlling for SWB at Time 1 (Sample 9)

	**Model 1 SWB**		**Model 2 Overall happiness**		**Model 3 Positive Affect**		**Model 4 Negative Affect**		**Model 5 Meaning in Life**	
	β	*p-value*	*β*	*p-value*	*β*	*p-value*	*β*	*p-value*	*β*	*p-value*
% Work	.19	.296	.37	.092	-.04	.855	-.3	.191	-.14	.529
% Overall leisure	.05	.733	-.1	.593	.17	.374	-.21	.283	.25	.172
% Active leisure	.41	.062	.79	.003	.59	.029	-.47	.094	.76	.003
% Passive leisure	-.17	.307	-.56	.006	-.14	.494	.02	.916	-.15	.456
% Necessities	.01	.843	.10	.023	-.01	.749	.04	.427	-.01	.813

Note. Work was captured with 3-items (work, study, commuting). Overall leisure was the sum of active and passive leisure. Active leisure was the sum of time spent praying/worshipping/meditating, socializing, exercising, intimate relations, going outdoors, hobbies. Passive leisure was the sum of time spent watching tv, napping/resting, relaxing, doing nothing. Necessities was measured as the sum of time spent shopping, personal hygiene, preparing food, doing housework For each composite, an episode weighted statistics was computed where the amount of time that respondents reported spending on each activity is weighted by the total amount of time spent in all measured activities: work, active leisure, passive leisure, necessities and other measured activities (phone/computer use, eating, waiting +other, social media use). The weighted time-use necessities were log transformed as skewness was greater than 3. Covariates were: age (range = 18 to 25), gender (female=1, male=0), race (dummy variables for being White, Black, Asian, Hispanic and Other), SES (education and income were entered as a composite or individually depending on the model), number of days since survey launch, number of co-habitants (range = 0 to 10).

Table S12i

Regression analyses predicting individual time-use differences at Time 1 by socio-demographic status (Sample 9)

	Gender (1 = female)	SES
**Commuting**	β = -.13, p = .324	β = -.07, p = .324
**Working**	β = .00, p = .393	β = .00, p = .122
**Learning**	β = .00, p = .723	β = .01, p = .002
**Praying**	β = -.10, p = .412	β = -.07, p = .289
**Socializing**	β = -.01, p = .929	β = .05, p = .277
**Exercising**	β = .000, p = .774	β = .00, p < .001
**Intimate**	β = -.14, p = .234	β = .01, p = 848
**Outdoor**	β = .00, p = .829	β = .00, p = .001
**Hobbies**	β = -.32, p < .001	β = -.03, p = .502
**Watching TV**	β = .02, p < .001	β = .01, p = .048
**Napping**	β = .01, p = .205	β = -.01, p < .001
**Relaxing**	β = .00, p = .468	β = 00, p = .975
**Doing nothing**	β = .00, p = .868	β = -.01, p = .002
**Shopping**	β = .00, p = .977	β = .01, p = .846
**Hygiene**	β = .09, p = .203	β = -.05, p = .246
**Preparing food**	β = .04, p = .537	β = -.02, p = .649
**Housework**	β = .00, p = .048	β = .00, p = .229
**Phone/Computer**	β = -.03, p = .001	β = .00, p = .492
**Eating**	β = .11, p = .036	β = .09, p = .001
**Waiting/Other**	β = .11, p = .303	β = .04, p = .445
**Social Media**	β = .02, p < .001	β = .00, p = .620

Note. Covariates were: age (range = 18 to 25), gender (female=1, male=0), race (dummy variables for being White, Black, Asian, Hispanic and Other), SES (education and income were entered as a composite or individually depending on the model), number of days since survey launch, number of co-habitants (range = 0 to 10).

Exploratory analyses with recalled pre-COVID-19 time-use

In the surveys of remote workers who were recruited primarily from the US and Brazil (n = 24,327), respondents also indicated what their time allocation looked like in a typical day prior to the pandemic. Below, we report results when controlling for these recalled pre-COVID-19 time-use measures. Additional results are present in Tables S13a-4c.

Sample 5

In models without covariates, gender differences in time-use did not hold when controlling for recalled pre-COVID-19 time-use. However, in models with our preregistered covariates, gender differences in time spent on necessities remained significant when controlling for these measures (M_{women} = 16.81, SD = 10.08 vs. M_{men} = 15.66, SD = 7.83, d = .12, p = .004; see Table S14a).

Exploratory analyses controlling for recalled pre-COVID-19 time-use showed a significant interaction between gender and parental status on overall leisure (F[1, 1,255] = 4.70, p = .030) and on necessities (F[1, 1,255] = 15.17, p < .001). Time spent on necessities increased for both mothers and fathers compared to non-mothers and non-fathers ($M_{mothers}$ = 23.84, SD = 10.93, vs. $M_{non\text{-}mothers}$= 13.56, SD = 7.75, d = 1.16, p < .001; $M_{fathers}$ = 18.98, SD = 7.51 vs. $M_{non\text{-}fathers}$ = 13.64, SD = 7.33, d = .72, p = .048). Simple slopes analyses showed that the differences in time spent on necessities *were* significant among parents (d = .53, p < .001) but not among non-parents (d = .01, p = .934), suggesting that mothers (vs. fathers) experienced an increase in time spent on necessities during COVID-19 (see Fig. S6, Panel A). See Table S14a for these exploratory analyses.

Sample 6

Unlike Sample 5 where gender differences in time-use during COVID-19 were no longer significant when controlling for recalled pre-COVID-19 time-use, in this study these exploratory analyses revealed that the following gender differences *were* significant: overall

leisure (M_{women} = 13.76, *SD* = 6.51 vs. M_{men} = 15.79, *SD* = 6.81; *d* = -.30, *p* < .001), active leisure (M_{women} = 4.40, *SD* = 3.79 vs. M_{men} = 5.62, *SD* = 4.40; *d* = -.30, *p* < .001), and necessities (M_{women} = 22.14, *SD* = 10.54 vs. M_{men} = 19.32, *SD* = 9.60; *d* = .28, *p* < .001), possibly due to having a larger sample size to detect such differences (see Table S14b).

Next, we ran exploratory analyses controlling for recalled pre-COVID-19 time-use and found a significant interaction between gender and parental status on overall leisure (*F*[1, 19,116] = 15.96, *p* < .001), passive leisure (*F*[1, 19,116] = 17.74, *p* < .001), and necessities (*F*[1, 19,116] = 39.80, *p* < .001). Time spent on overall leisure decreased for both mothers and fathers compared to non-mothers and non-fathers ($M_{mothers}$ = 11.63, *SD* = 6.03 vs. $M_{non\text{-}mothers}$ = 15.15, *SD* = 15.15, *d* = -.56, *p* < .001; $M_{fathers}$ = 14.25, *SD* = 6.25 vs. $M_{non\text{-}fathers}$ = 16.95, *SD* = 6.99, *d* = -.40, *p* = .048). The differences in time-use were significant among non-parents (*d* = -.15, *p* < .001) and parents (*d* = -.43, *p* = .934), suggesting that women, and especially mothers, experienced a decrease in time spent on overall leisure during COVID-19. These differences hold for our preregistered covariates.

Regarding passive leisure differences, both mothers and fathers spent less time pursuing passive leisure activities ($M_{mothers}$ = 7.74, *SD* = 4.62 vs. $M_{non\text{-}mothers}$ = 10.42, *SD* = 5.30, *d* = -.53, *p* < .001; $M_{fathers}$ = 9.07, *SD* = 4.80 vs. $M_{non\text{-}fathers}$ = 11.01, *SD* = 5.80, *d* = -.36, *p* = .004). While these differences in time-use were not significant among non-parents (*d* = -.11, *p* = .915), they *were* among parents (*d* = -.28, *p* < .001).

In terms of necessities and similar to Sample 5 results, both mothers and fathers spent more time on necessities ($M_{mothers}$ = 26.52, *SD* = 11.05 vs. $M_{non\text{-}mothers}$ = 19.28, *SD* = 9.13, *d* = .73, *p* < .001; $M_{fathers}$ = 22.13, *SD* = 9.67 vs. $M_{non\text{-}fathers}$ = 17.21, *SD* = 8.99, *d* = .53 *p* < .001). The differences in time-use were significant among non-parents (*d* = .23, *p* < .001) and among parents (*d* = .42, *p* < .001), suggesting that while both parents spent more time on

necessities during the pandemic than before, mothers might have experienced a greater increase in time spent on necessities (see Fig. S6, Panel B).

Sample 7

In this study, gender differences in time-use were not significant when running exploratory analyses controlling for the recalled pre-COVID-19 time-use (see Table S11h). Next, we ran exploratory analyses controlling for these recalled time-use measures. Unlike Studies 5-6 results, the interaction between gender and parental status on necessities was not significant even in models with covariates (F[1, 729] = .114, p = .735), suggesting that in this sample the potential increase in time spent on necessities did not differ by gender among parents, possibly due to the diversity of countries included in this sample. See Table S14c for detailed results.

Table S13a

Descriptive statistics for recalled pre-COVID time-use and mean differences with during COVID time-use (Sample 5)

	**During-COVID Time-use**		**Recalled Pre-COVID Time-Use**		**Δ between pre and during COVID**	**Two-tailed t-value**	**p-value**
	**Mean**	**SD**	**Mean**	**SD**			
Work [a]	1.57%	.12	1.60%	.12	-.03	-14.76	< .001
Model with covariates:	1.56%	.12	1.59%	.12	-.03	-	< .001
Overall leisure	15.99%	6.99	15.29%	7.12	.70	4.75	< .001
Model with covariates:	16.52%	6.95	15.52%	6.98	-.66	-	< .001
Active leisure	6.75%	4.22	6.95%	4.66	-.20	-1.67	.095
Model with covariates:	6.72%	4.09	6.98%	4.52	-.26	-	.046
Passive leisure	9.38%	5.68	8.44%	5.31	.93	8.01	< .001
Model with covariates:	9.57%	5.75	8.62%	5.32	.94	-	< .001
Necessities	16.47%	9.34	14.40%	8.05	2.07	12.34	< .001
Model with covariates:	16.39%	9.28	14.28%	7.89	2.10	-	< .001

Note. For models without covariates we ran paired t-tests. For models with covariates we ran repeated measures models with time as a within-subjects factor. Descriptive statistics for the time-use measures are reported as episode-weighted statistics (i.e. the percentage of time that respondents reported spending on each activity is weighted by the total amount of time they spent in all other activities measured within that sample). Work is a composite of 2 items (i.e. working productively, working unproductively); overall leisure is a composite of 1 item capturing active leisure like going outdoors, and 1 item capturing passive leisure like watching TV. Necessities is a composite of 2 items (i.e. doing errands/household chores and taking care of/spending time with family). Overall leisure is a composite of active and passive leisure. [a] Given that the skewness value of the time-use work composite was above 8.32 (thus above the pre-registered cutoff point of 2), we log transformed this variable.

Table S13b

Descriptive statistics for recalled pre-COVID time-use and mean differences with during COVID time-use (Sample 6)

	During-COVID Time-use		Recalled Pre-COVID Time-use		Δ between pre and during COVID	Two-tailed t-value	p-value
	**Mean**	**SD**	**Mean**	**SD**			
Work	34.94%	9.42	36.70%	8.98	-1.76	-34.72	< .001
Model with covariates:	34.85%	9.34	36.67%	8.88	-1.83		< .001
Overall leisure	14.62%	6.81	16.05%	7.02	-1.43	-35.94	< .001
Model with covariates:	14.65%	6.77	16.04%	6.96	-1.40		< .001
Active leisure	5.00%	4.21	7.14%	8.84	-2.14	-63.85	< .001
Model with covariates:	5.00%	4.16	7.13%	4.80	-2.13		< .001
Passive leisure	9.78%	5.39	9.01%	5.05	.77	27.43	< .001
Model with covariates:	9.80%	5.39	9.02%	5.05	.79		< .001
Necessities	20.87%	10.27	17.62%	9.13	3.25	66.06	< .001
Model with covariates:	20.82%	10.26	17.54%	9.07	3.29		< .001

Note. For models without covariates we ran paired t-tests. For models with covariates we ran repeated measures models with time as a within-subjects factor. Descriptive statistics for the time-use measures are reported as episode-weighted statistics (i.e. the percentage of time that respondents reported spending on each activity is weighted by the total amount of time they spent in all other activities measured within that sample). Work is a composite of 2 items (i.e. working productively, working unproductively); overall leisure is a composite of 1 item capturing active leisure like going outdoors, and 1 item capturing passive leisure like watching TV. Necessities is a composite of 2 items (i.e. doing errands/household chores and taking care of/spending time with family). Overall leisure is a composite of active and passive leisure.

Table S13c

Descriptive statistics for recalled pre-COVID time-use and mean differences with during COVID time-use (Sample 7)

	**During-COVID Time-use**		**Recalled Pre-COVID Time-use**		**Δ between pre and during COVID**	**Two-tailed t-value**	**p-value**
	**Mean**	**SD**	**Mean**	**SD**			
Work	35.95%	9.73	37.43%	8.85	-1.48	-6.12	< .001
Model with covariates:	36.06%	9.70	37.65%	8.60	-1.59		< .001
Overall leisure	15.80%	7.43	16.40%	7.10	-.60	-3.22	.001
Model with covariates:	15.63%	7.22	16.17%	6.76	-.54		.009
Active leisure	5.86%	4.54	7.47%	5.11	-1.61	-9.89	< .001
Model with covariates:	5.71%	4.39	7.28%	4.87	-1.58		< .001
Passive leisure	10.06%	5.87	9.00%	5.19	1.07	7.10	<.001
Model with covariates:	10.04%	5.75	8.96%	5.08	1.08		< .001
Necessities	18.32%	10.14	15.89%	9.06	2.43	11.07	< .001
Model with covariates:	18.29%	10.14	15.84%	9.15	2.45		< .001

Note. For models without covariates we ran paired t-tests. For models with covariates we ran repeated measures models with time as a within-subjects factor. Descriptive statistics for the time-use measures are reported as episode-weighted statistics (i.e. the percentage of time that respondents reported spending on each activity is weighted by the total amount of time they spent in all other activities measured within that sample). Work is a composite of 2 items (i.e. working productively, working unproductively); overall leisure is a composite of 1 item capturing active leisure like going outdoors, and 1 item capturing passive leisure like watching TV. Necessities is a composite of 2 items (i.e. doing errands/household chores and taking care of/spending time with family). Overall leisure is a composite of active and passive leisure.

Table S14a

Regression analyses predicting time-use differences in necessities by gender and parental status controlling for recalled pre-COVID-19 time-use (Sample 5)

Gender (1 = female)	Δ = 1.01, p = .046
Model with covariates:	Δ = 1.96, p < .001
Models with recalled pre-covid-19 time-use	Δ = .56, p = .108
Models with recalled pre-covid-19 time-use & covariates	Δ = 1.04, p = .004
Parental status (1 = yes)	Δ = 7.80, p < .001
Model with covariates:	Δ = 7.77, p < .001
Models with recalled pre-covid-19 time-use	Δ = 3.53, p < .001
Models with recalled pre-covid-19 time-use & covariates	Δ = 2.60, p < .001
**Gender** (1=female) X **Parental status** (1=yes)	b = 4.96; p < .001
Model with covariates:	b = 5.14; p < .001
Models with recalled pre-covid-19 time-use	b = 2.58; p < .001
Models with recalled pre-covid-19 time-use & covariates	b = 2.96; p < .001

Note. Covariates vary by model depending on predictors entered in each model: age, gender, education, monthly household income, household size, number of children (under 18), and number of days since survey launch. Age, monthly household income, education, number of children (under 18), and number of days since survey launch were entered as continuous variables. Gender (1=female) was dummy coded. In this study, household size was measured as a binary variable (1=living with others; 0=living alone). All respondents in this dataset were employed adults. We did not capture weekly work hours in this dataset.

Table S14b

Regression analyses predicting time-use differences in necessities by gender and parental status controlling for recalled pre-COVID-19 time-use (Sample 6)

Gender (1 = female)	Δ = 2.81, p < .001
Model with covariates:	Δ = 3.35, p < .001
Models with recalled pre-covid-19 time-use	Δ = 1.54, p < .001
Models with recalled pre-covid-19 time-use & covariates	Δ = 1.85, p < .001
Parental status (1 = yes)	Δ = 5.84, p < .001
Model with covariates:	Δ = 7.73, p < .001
Models with recalled pre-covid-19 time-use	Δ = 2.64, p < .001
Models with recalled pre-covid-19 time-use & covariates	Δ = 1.69, p < .001
**Gender** (1=female) X **Parental status** (1=yes)	b = 2.24; p < .001
Model with covariates:	b = 2.55; p < .001
Models with recalled pre-covid-19 time-use	b = 1.15; p < .001
Models with recalled pre-covid-19 time-use & covariates	b = 1.35; p < .001

Note. Covariates vary by model depending on predictors entered in each model: age, gender, education, monthly household income, household size, number of children (under 18), and number of days since survey launch. Age, monthly household income, education, number of children (under 18), and number of days since survey launch were entered as continuous variables. Gender (1=female) was dummy coded. In this study, household size was measured as a binary variable (1=living with others; 0=living alone). All respondents in this dataset were employed adults. We did not capture weekly work hours in this dataset.

Table S14c

Regression analyses predicting time-use differences in necessities by gender and parental status controlling for recalled pre-COVID-19 time-use (Sample 7)

Gender (1 = female)	$\Delta = 2.45$, $p < .001$
Model with covariates:	$\Delta = 2.48$, $p < .001$
Models with recalled pre-covid-19 time-use	$\Delta = .65$, $p = .140$
Models with recalled pre-covid-19 time-use & covariates	$\Delta = 88$, $p = .067$
Parental status (1 = yes)	$\Delta = 8.14$, $p < .001$
Model with covariates:	$\Delta = 6.51$, $p < .001$
Models with recalled pre-covid-19 time-use	$\Delta = 3.95$, $p < .001$
Models with recalled pre-covid-19 time-use & covariates	$\Delta = 2.25$, $p = .014$
**Gender** (1=female) X **Parental status** (1=yes)	$b = 3.31$; $p = .007$
Model with covariates:	$b = 2.83$; $p = .031$
Models with recalled pre-covid-19 time-use	$b = .20$; $p = .919$
Models with recalled pre-covid-19 time-use & covariates	$b = .15$; $p = .868$

Note. $N = 933$. Covariates vary by model depending on predictors entered in each model: age, gender, education, monthly household income, household size, number of children (under 18), and number of days since survey launch. Age, monthly household income, education, number of children (under 18), and number of days since survey launch were entered as continuous variables. Gender (1=female) was dummy coded. In this study, household size was measured as a binary variable (1=living with others; 0=living alone). All respondents in this dataset were employed adults. We did not capture weekly work hours in this dataset.

Fig. S6 | Interaction between gender and parental status on time spent on necessities during COVID-19

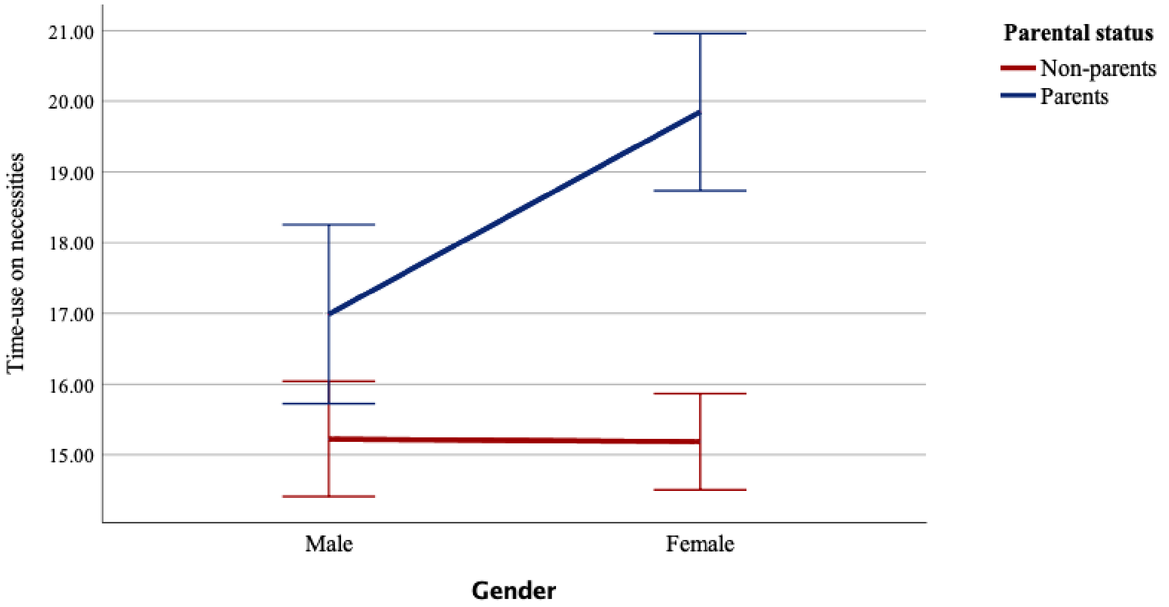

Panel A

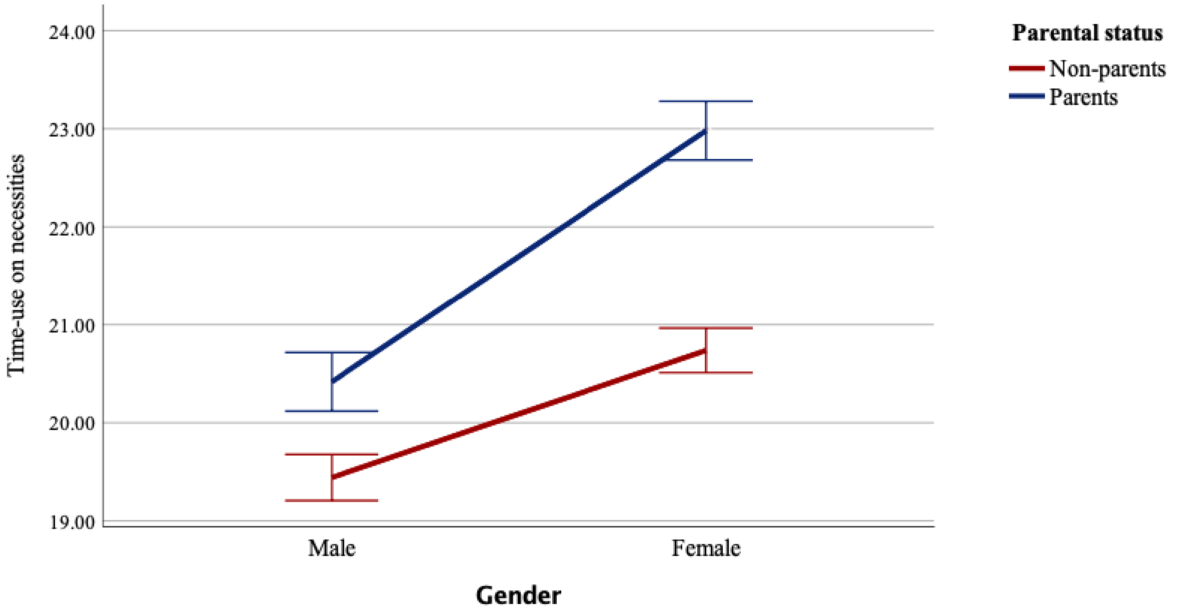

Panel B

Note. In these analyses we control for recalled time spent on necessities pre-COVID-19 as well as for ur preregistered covariates: age, income, number of children, education, number of people in the household, and days since survey launch. Necessities is a composite of household chores and taking care of others. Error bars are $CI_{95\%}$. Panel A is the pattern observed in Sample 5 and Panel B is the patterned observed in Sample 6.

Additional Analyses Sample 4: Shared Time on Chores and Caretaking Responsibilities

Additional measures. As part of a different project, in Sample 4 respondents who indicated living with someone else as a couple were also asked to report: 1. How many hours did they and their partner spent together on household tasks, 2. How many hours did they spend on household chores alone without their partner, and 3. How many hours did their partner spend on household chores alone without them (slide scale from 0 to 100, with max 100 across all three questions).

In addition, among these respondents those who further indicated having children were asked to report: 1. How many hours did they and their partner spend together on caretaking tasks, 2. How many hours did they spend on caretaking tasks alone without their partner, and 3. How many hours did their partner spend on caretaking tasks alone without them (slide scale from 0 to 100, with max 100 across all three questions).

Additional results. We followed the same procedures as for these additional time-use measures. In line with our pre-registered steps for our main analyses, we regressed gender on these time-use outcomes. These exploratory analyses revealed no gender differences in amount of time spent together on household tasks or caretaking tasks. However, these analyses showed a consistent pattern whereby women (vs. men) reported spending more time alone than their partner on both household and caretaking (see Figure S below). Similarly, men (vs. women) reported that their partner spent more hours alone completing both household and caretaking tasks (see Figures S).

Next, we regressed happiness on the household time-use measures and on the caretaking time-use measures respectively and found that spending time together on both types of necessities was positively associated with happiness. By contrast, completing either of these necessities alone was negatively associated with happiness. Notably, there was no association between how many hours one's partner spends on caretaking responsibilities and happiness (see Table S15 below).

Fig. S7a | Gender differences in time spent alone or with partner on household tasks (Sample 4).

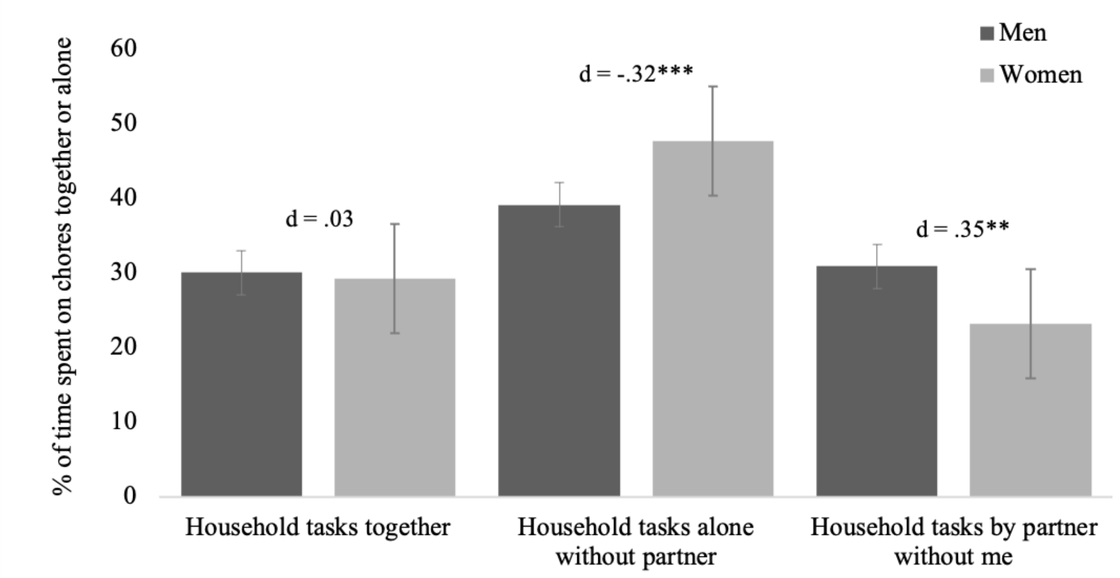

Note. N = 520 for items capturing time spent on household tasks. N = 340 for items capturing time spent on caretaking. Covariates: age, gender, employment status, education, monthly household income, household size, number of children, and number of days since survey launch. Age, monthly household income, education, household size, number of children, weekly work hours, and number of days since survey launch were entered as continuous variables. Gender (1=female) and employment status (1 = employed) were dummy coded.

Fig. S7b | Gender differences in time spent alone or with partner on caretaking tasks (Sample 4).

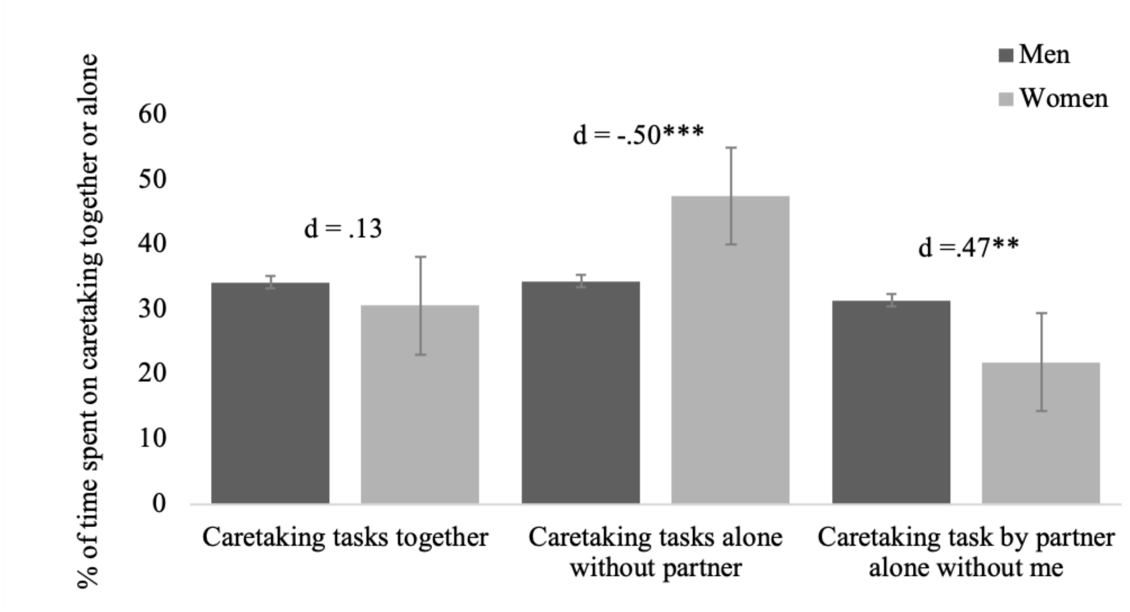

Note. N = 520 for items capturing time spent on household tasks. N = 340 for items capturing time spent on caretaking. Covariates: age, gender, employment status, education, monthly household income, household size, number of children, and number of days since survey launch. Age, monthly household income, education, household size, number of children, weekly work hours, and number of days since survey launch were entered as continuous variables. Gender (1=female) and employment status (1 = employed) were dummy coded.

Table S15
Additional analyses of time-use predicting happiness (Sample 4)

	Models without covariates					Models with covariates				
	**β**	**SE**	**t**	**p**	**r-partial**	**β**	**SE**	**t**	**p**	**r-partial**
Household tasks together	.23	.043	5.50	<.001	.24	.22	.055	3.97	<.001	.21
Household tasks alone without partner	-.13	.044	-2.85	.005	-.13	-.11	.055	-1.89	.059	-.01
Household tasks partner without me	-.14	.044	-3.18	.002	-.14	-.13	.055	-2.45	.015	-.13
Caretaking tasks together	.18	.054	3.28	.001	.18	.17	.055	3.06	.002	.17
Caretaking tasks alone without partner	-.15	.054	-2.76	.006	-.15	-.14	.055	-2.53	.012	-.14
Caretaking tasks partner alone without me	-.05	.054	-.90	.370	-.05	-.06	.056	.99	.320	-.05

Notes. N = 520 for items capturing time spent on household tasks. N = 340 for items capturing time spent on caretaking. Covariates: age, gender, employment status, education, monthly household income, household size, number of children, and number of days since survey launch. Age, monthly household income, education, household size, number of children, weekly work hours, and number of days since survey launch were entered as continuous variables. Gender (1=female) and employment status (1 = employed) were dummy coded.
